# Disentangling juxtacrine from paracrine signalling in dynamic tissue

**DOI:** 10.1371/journal.pcbi.1007030

**Published:** 2019-06-13

**Authors:** Hiroshi Momiji, Kirsty L. Hassall, Karen Featherstone, Anne V. McNamara, Amanda L. Patist, David G. Spiller, Helen C. Christian, Michael R. H. White, Julian R. E. Davis, Bärbel F. Finkenstädt, David A. Rand

**Affiliations:** 1 Zeeman Institute for Systems Biology & Infectious Disease Epidemiology Research, University of Warwick, Coventry, United Kingdom, Mathematics Institute, University of Warwick, Coventry, United Kingdom; 2 Department of Statistics, University of Warwick, Coventry, United Kingdom; 3 Faculty of Biology, Medicine & Health, University of Manchester, Manchester, United Kingdom; 4 Systems Microscopy Centre, University of Manchester, Manchester, United Kingdom; 5 Department of Physiology, Anatomy and Genetics, University of Oxford, Oxford, United Kingdom; University of Edinburgh, UNITED KINGDOM

## Abstract

Prolactin is a major hormone product of the pituitary gland, the central endocrine regulator. Despite its physiological importance, the cell-level mechanisms of prolactin production are not well understood. Having significantly improved the resolution of real-time-single-cell-GFP-imaging, the authors recently revealed that prolactin gene transcription is highly dynamic and stochastic yet shows space-time coordination in an intact tissue slice. However, it still remains an open question as to what kind of cellular communication mediates the observed space-time organization. To determine the type of interaction between cells we developed a statistical model. The degree of similarity between two expression time series was studied in terms of two distance measures, Euclidean and geodesic, the latter being a network-theoretic distance defined to be the minimal number of edges between nodes, and this was used to discriminate between juxtacrine from paracrine signalling. The analysis presented here suggests that juxtacrine signalling dominates. To further determine whether the coupling is coordinating transcription or post-transcriptional activities we used stochastic switch modelling to infer the transcriptional profiles of cells and estimated their similarity measures to deduce that their spatial cellular coordination involves coupling of transcription via juxtacrine signalling. We developed a computational model that involves an inter-cell juxtacrine coupling, yielding simulation results that show space-time coordination in the transcription level that is in agreement with the above analysis. The developed model is expected to serve as the prototype for the further study of tissue-level organised gene expression for epigenetically regulated genes, such as prolactin.

## Author summary

Prolactin is a major hormone product of the pituitary gland, the central endocrine regulator found underneath the brain. In mammals it is crucial for milk production and reproductive function. Production of such an important protein needs to be regulated tightly, and therefore one might imagine that its gene expression is largely static. However, recent experiments using real-time imaging techniques at a single-cell resolution have revealed prolactin gene transcription to be highly dynamic and stochastic in nature, while displaying clear tissue-scale space-time coordination. This discovery raised a new question, namely, what kind of cellular communication mediates such a space-time organization? In this study, by developing a statistical method that involves network theory, we show that such unexpected behaviour involves contact-driven inter-cell signalling. The study develops a mathematical model that can reproduce realistic levels of space-time coordinated gene expression. The method and model developed here are generic and can be used in the study of other signalling systems that show space-time coordinated behaviour.

## Introduction

**Fig 1:**
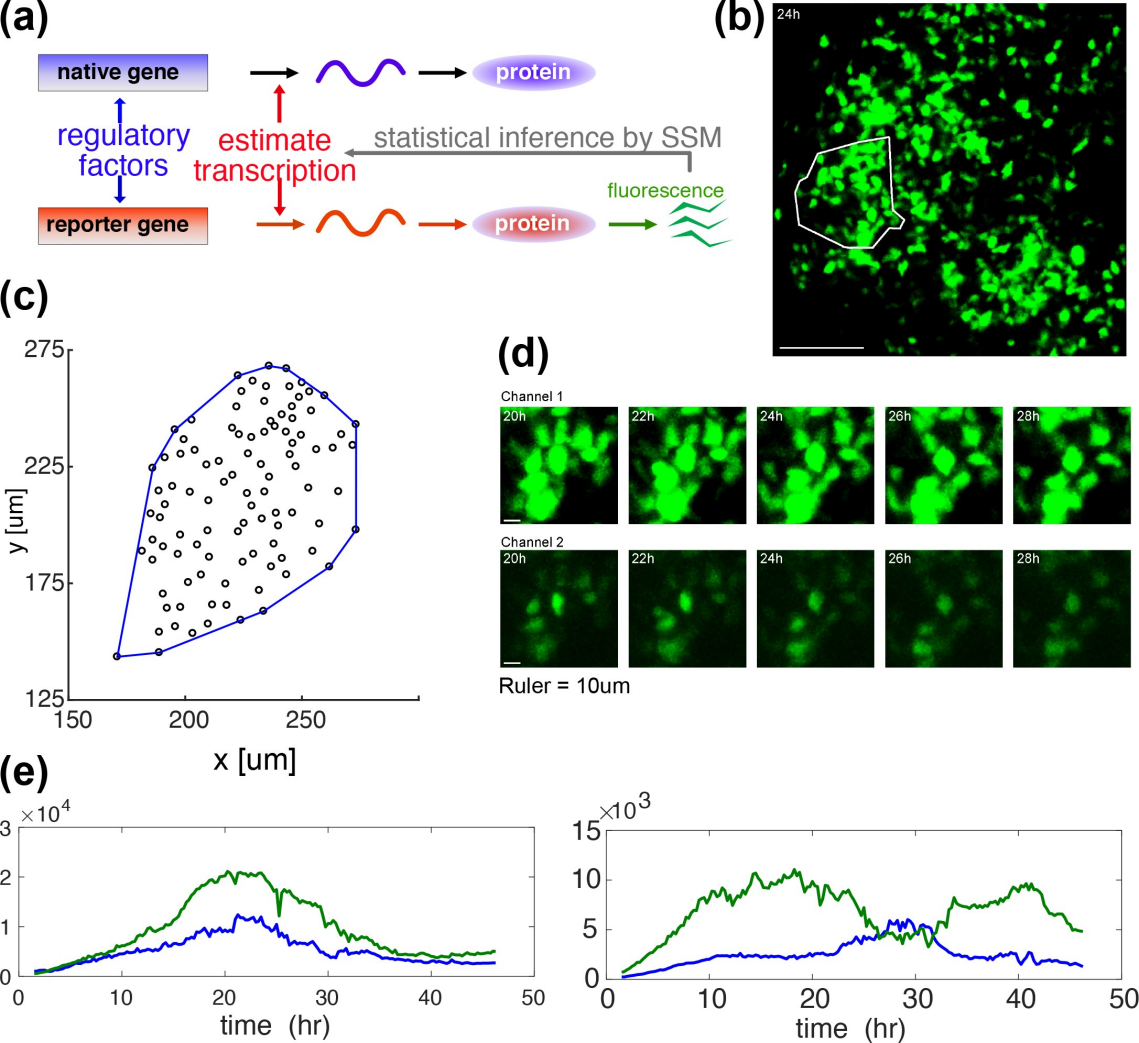
GFP imaging in space and time–measurement and representations of prolactin gene transcription. **(a)** A diagrammatic illustration of how the GFP reporter measurement is associated with the underlying process of native prolactin gene expression. Expression of the d2EGFP reporter transgene in these studies is controlled by a fragment of the human prolactin gene locus (as described previously[1, 8]). Both the endogenous prolactin gene and the prolactin-d2EGFP reporter gene mRNAs are transcribed in parallel, but are then translated independently into respective proteins (reproduced from [10]). **(b)** A GFP image of an intact tissue slice from an adult male prolactin-d2EGFP transgenic reporter rat, with a white enclosure to indicate a cell-tracked area for analysis. **(c)** A spatial distribution of cell centroids, defined as the median over the time-course of the coordinates, with its convex hull (blue). There are 101 cells. The convex hull will be used in Fig 3A in the estimation of the mean cell size. **(d)** Magnified typical video-frame sequences in time, showing dynamic changes in reporter gene transcription over a collection of single cells. A single sample is recorded concurrently in two channels of high (upper row) and low (lower row) sensitivities. **(e)** Typical GFP signals in a time course, showing high (left) and low (right) correlations. Correlation coefficients are 0.96 (left) and 0.14 (right) respectively. Each time series obtained in a single cell is reconstructed from two recordings illustrated in (d). See Fig 2B for the reconstruction process.

**Fig 2:**
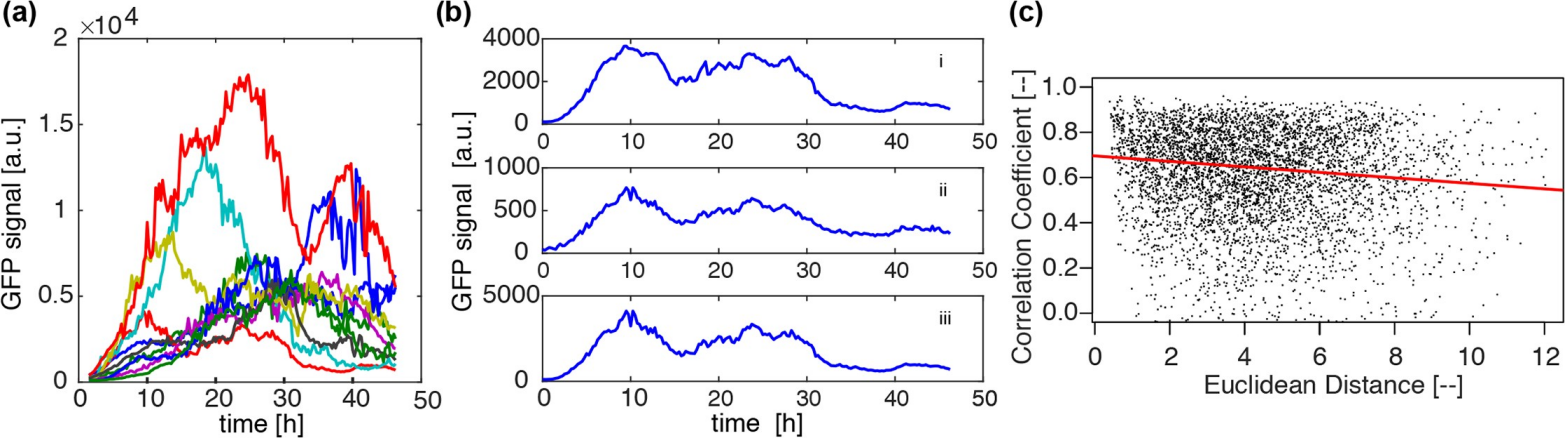
Properties in network-free analysis in space and time. **(a)** GFP time series of ten randomly chosen cells. Time series are reconstructed from two GFP signals recorded simultaneously with different sensitivities to account for saturation effects and to maximise the observed dynamic range. **(b)** An example of GFP signal reconstruction. Signals from channels 1 (i) and 2 (ii) are combined to restore a linearity between light intensity and GFP reading, yielding a reconstructed signal as in (iii). See Materials and Methods for detailed protocols. **(c)** Correlation coefficients (Pearson) between GFP time series plotted against Euclidean distance for all cell pairs. The red line shows fit of median regression. Here and throughout the present paper Euclidean distance is normalised by the median cell diameter (see Fig 3A), and is therefore dimensionless. A significant rightward decline, with corresponding *p*-values shown in S1 Table, is indicative of the presence of space-time coordination in prolactin gene expression profiles.

Gene expression at a single-cell level is highly dynamic in time, and the processes involved in gene activation and inactivation are now well-known to be highly stochastic [1–7]. The use of single-cell live imaging techniques, which employ luciferase or light emitting proteins such as destabilised EGFP as a reporter (Fig 1A, [1, 2]) aided by statistical models that infer the gene transcription process from reporter signals, has been critical for this development in the understanding of transcriptional dynamics. While they were thought to be smoothly changing graded processes, mathematical modelling has indicated that they can be well explained by discrete time stochastic switch functions [1, 3–6]. This might be a binary switching between on and off states [1], or a more complex process [7–9]. While much progress has been made in understanding the processes involved in transcriptional control in single cells, an important challenge is to translate this to understanding the transcriptional dynamics in multicellular tissues. In the context of the behaviour of intact tissue, overall gene expression levels should be accurately controllable and predictable, but it is still unclear how overall coordinated tissue function emerges from the switching transcriptional dynamics of individual cells.

Space-time coordination requires communication between cells. At the tissue scale we consider such signalling to be either juxtacrine or paracrine ([11], Ch. 15). Juxtacrine signalling is a type of cellular communication between contacting cells, for example by means of gap junctions that allow for signalling molecules to pass from cell to cell. This type of interaction can be transitive, allowing distant cells to communicate with each other by successive cellular contacts. In contrast to this, paracrine signalling does not rely on any cellular contact but depends on the intercellular diffusion of signalling molecules. It is possible that these two signalling mechanisms coexist.

The mammalian pituitary gland, which secretes a series of key hormones including prolactin, has proved to be an excellent model system for the study of dynamic gene expression *in vivo* ([1, 8, 12]). In contrast to tissues with deterministic developmental programmes, the pituitary gland generates complex function from an assortment of intermingled cells that require both short- and long-term adaptive processes. The different cell types are arranged as interdigitated networks, linked by specific cell-contacts, including gap junctions [13]. The cell networks adapt structurally and functionally to ensure temporal optimisation of hormone expression in response to different reversible physiological adaptations such as pregnancy and lactation [14].

In the present paper, we propose a novel statistical methodology to assess the existence of juxtacrine and paracrine signalling mechanisms between cells in the pituitary gland by analysing quantitative single-cell live imaging data obtained from rat pituitary tissue slices. We apply this method to the transcriptional dynamics of the prolactin gene after inferring dynamical transcription profiles from imaging using Stochastic Switch Modelling (SSM, Fig 1A, [7]). By developing a stochastic simulation model, we test if the signalling mechanisms identified above are sufficient to reproduce the observed space-time structure.

## Results

While we studied three adult tissue portions, labelled D1, D2, and D3, to ensure reproducibility, we present mainly the results obtained for D1, which correspond to the data studied in [8]. The results for D2 and D3 are consistent with the findings for D1, and are summarised in S1–S3 Tables.

Fig 1B shows a snapshot during the GFP-imaging of one of the tissue slices studied here, with a white enclosure indicating the analysed area. The tissue areas consist of about 100 cells, whose spatial locations are found to be random (Fig 1C, examined in detail in [8]), while their transcriptional behaviour appears quite dynamic (Fig 1D). Some cell pairs display high correlation in their GFP time series, but others do not (Fig 1E). In [8], we showed that the expression of the prolactin gene was coordinated in space and time at a tissue scale in adult male rats, while no such coordination was observed at an embryonic or a neonatal stage. However, this is not merely a consequence of a purely spatial organisation of cells growing in development. While such structures have been documented for the pituitary on a relatively large scale [12], we showed in [8] that the cellular locations were statistically random in space in our adult tissues while the space-time coordinated behaviour was underlain by the cellular network defined by the cellular contacts. The importance of gap junctions for this coordination was suggested because the application of the gap-junction inhibitor, AGA (Alpha-glycyrrhetinic acid), significantly reduced the correlation between transcriptional time-profiles of individual cells. This motivated our development of the method proposed here to estimate the extent of juxtacrine and paracrine signalling.

### Space-time correlation is evident in a network-free analysis

While we have revealed the presence of space-time structure in the set of GFP signals on prolactin in our previous work [8], here we examine this important property in more detail, and later provide a mathematical model for it. Fig 2A shows ten randomly chosen GFP time series, for visual clarity, from all 101 cells in the tissue of interest. These are carefully reconstructed profiles by combining the signals from two channels of different light sensitivity such that the linearity holds between light intensity and the GFP population (Fig 2B, see also Materials and Methods). Pearson’s correlation coefficients are calculated between all cell pairs, and plotted against the corresponding Euclidean distances in Fig 2C. To analyse a trend in this kind of scatter plot we generally used the method of quantile regression [15], which is robust to the non-normality arising from the boundedness of the correlation coefficient. In particular, we focused on the median (red). A statistically significant rightward decline in this median regression line indicates the presence of a space-time correlation structure (see S1 Table for the corresponding *p*-value). Here, and throughout this paper, we use the one-sided t-test.

**Fig 3:**
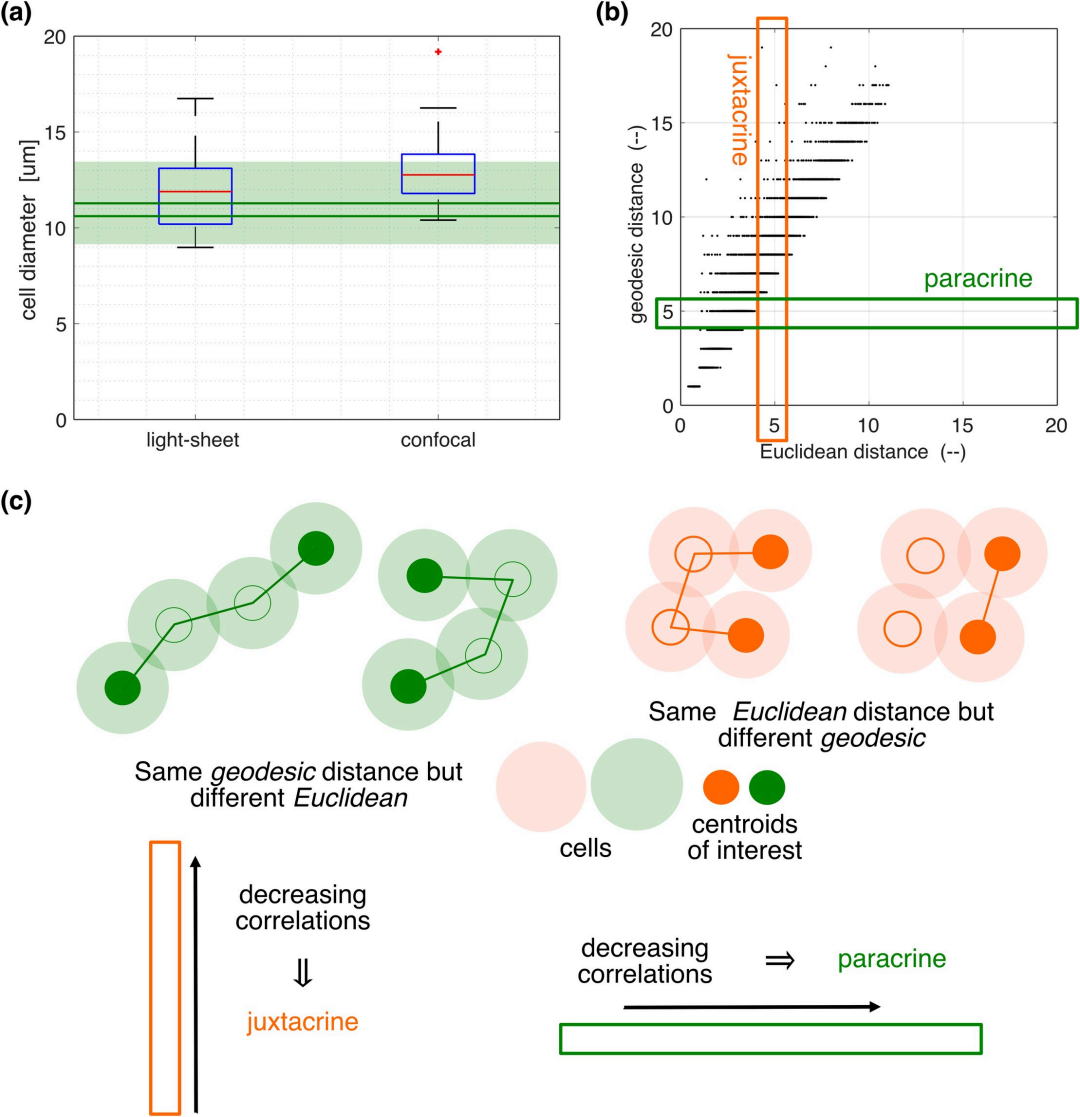
Cellular network estimation and characterisation of cell pairs by two distance measures. **(a)** Box-plots of cell size distributions obtained from light-sheet (*left*), and confocal (*right*) micrographs. A red + symbol is an outlier found above the whisker, which is defined to be q3 ± 1.5 (q3—q1), where q1 and q3 are the respective 25th and 75th percentiles. The results are compared to the estimations obtained from the spatial distributions of cell centroids as in Fig 1C (*green*), with details described in Materials and Methods. With three replicated datasets, the solid horizontal lines represent the means of upper and lower bounds, whose precise definitions are given in Materials and Methods, while the shade is defined by the maximum of the upper bounds and the minimum of the lower bounds. **(b)** The relationship between Euclidean and geodesic distances for all cell pairs (here and throughout the present paper, Euclidean distance is normalised by the median cell diameter). The latter is a network distance measure, and is defined in detail in Materials and Methods. **(c)** Schematic illustrations of the cell pairs that are subject to juxtacrine (*orange*) and paracrine (*green*) signalling. The cellular network is materialised by their physical contacts. In the orange diagram, geodesic distances are different, 3 on the left and 1 on the right, while Euclidean distances are similar. In the green diagram Euclidean distances are different, larger on the left and small on the right, while the geodesic distance is 3 in both cases. For more details about these two distances, see Materials and Methods (see subsection on Spatial properties in the Computational section). A decreasing correlation with geodesic (Euclidean) distance in a sub-population of cell pairs having a similar Euclidean (geodesic) distance, as illustrated by the enclosed thin orange vertical (green horizontal) rectangle in (b), implies juxtacrine (paracrine) signalling.

### Cellular network estimation allows identification of signalling types

While the results in Fig 2C establish the presence of a space-time correlation, they do not reveal underlying mechanisms. To identify the contributions of juxtacrine and/or paracrine signalling, we introduce another distance measure, namely geodesic distance. Geodesic distance between two cells is associated with their shortest paths defined on a cellular network, which in turn is defined here by cellular contacts [16]. It is the smallest number of cellular contacts between two cells, and therefore takes an integer value if they reside on a common cluster, or is set to infinity if there is no path between them.

**Fig 4:**
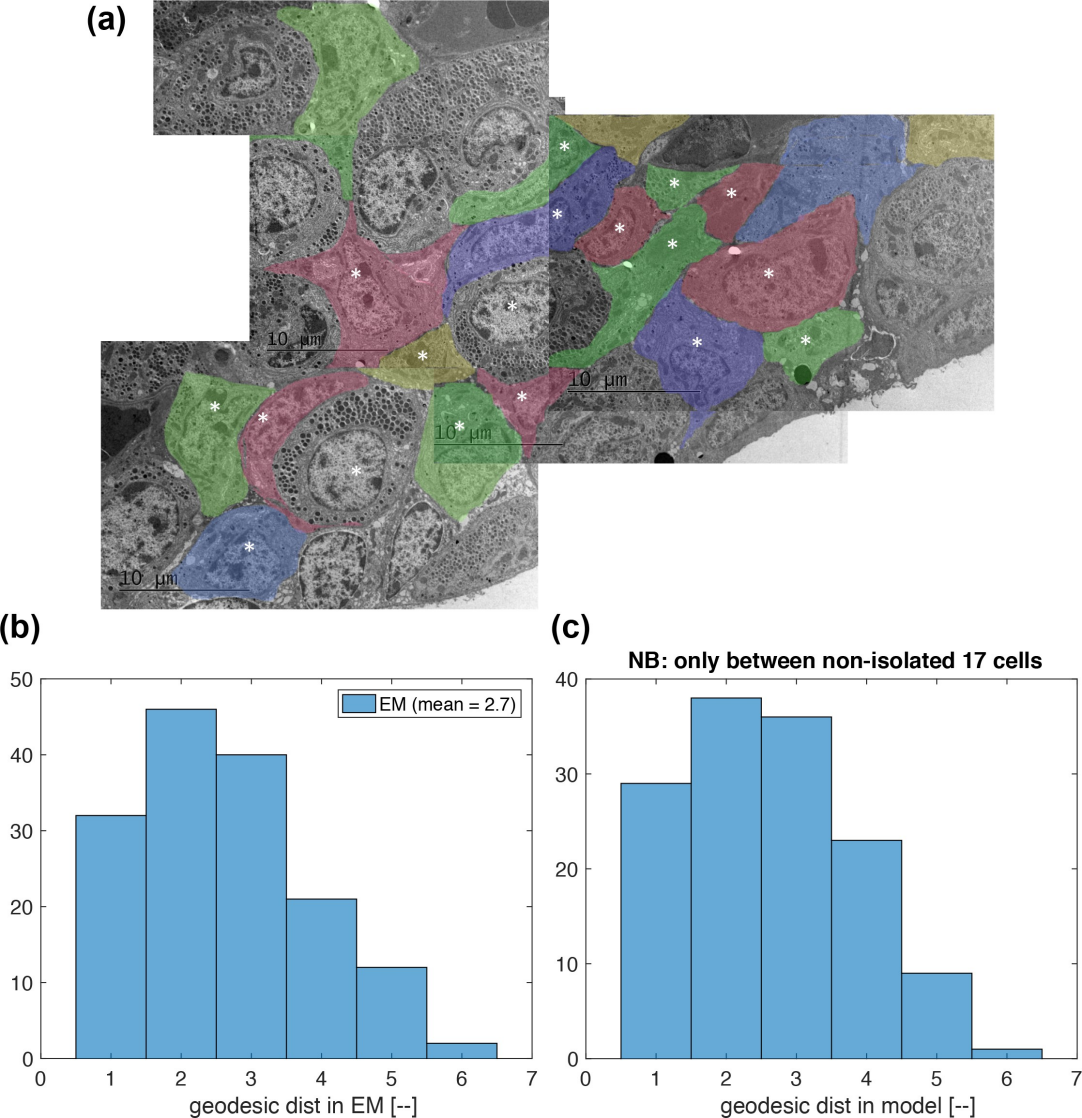
Distributions of geodesic distance compared between an electron microscopic (EM) image and the model applied to a GFP image. **(a)** A montage of x800 EM images of anterior pituitary tissue, which is different from those GFP imaged (D1, D2, D3). Each lactotroph is shaded in a different colour to distinguish individual cells. There are 18 cells that are fully contained in this montage, and are labelled by *. **(b)** The distribution of geodesic distance estimated in the EM picture in (a). The 18 cells labelled by * are examined. **(c)** The distribution of geodesic distance estimated in the model with a GFP dataset. The 18 cells nearest to the tissue centre are examined (see Fig 1C). One cell was found to be isolated from the other 17 cells, causing the histogram y-axis limit smaller than that in (b).

The geodesic distances are estimated as detailed in Materials and Methods. Although we cannot directly observe contacts between cells, we can use cell positions and sizes to statistically estimate geodesic distance. It is therefore important to identify the distribution of cell sizes. Fig 3A shows these distributions inferred from two experimental methods–light-sheet microscopy, and confocal microscopy. They both result in similar values for the mean and standard deviation of the cell diameters, namely (12.0, 2.2) μm and (13.1, 1.9) μm, respectively. Because light-sheet microscopy allows for measurement in the depth as well as the horizontal directions, it can be assumed to give more precise measurements, and is therefore used in our further analysis, while the cell-size sensitivities are investigated in S3 Table. We also considered electron microscopic measurement ([8], Fig 4), but this resulted in apparently small sizes (9.0, 1.2) μm due to some cell shrinkage during the fixation process [17, 18]. Electron microscopic estimates of cell sizes were therefore not used in this study.

In addition to the above investigations, cell sizes are also estimated computationally from the spatial distributions of cell centroids (see Fig 1C and Materials and Methods). In Fig 3A, the estimated mean cell diameters (green) in three datasets, D1, D2 and D3, are compared to the other experimentally determined values. A good degree of coincidence between them suggests that experimental estimates are reasonable. It also suggests that cells are densely packed, as the computational cell size estimation is based on a model that assumes a 2-D dense packing of circles that accounts for 79% of the tissue area (see Materials and Methods).

Using the cell size distribution obtained from the light-sheet microscopy, geodesic distances between all cell pairs are statistically estimated, and compared to their corresponding Euclidean distances in Fig 3B. While there is an overall linear relationship between these two distance pairs, there is still some variation, which allows us to distinguish between the two types of signalling as follows. All cell pairs, with associated GFP time series, are now characterised by three quantities (*r*, *d*_E_, *d*_G_), with *r* the correlation coefficient, *d*_E_ Euclidean distance, and *d*_G_ geodesic distance. In a collection of cell pairs within a given *d*_E_ range, if *r* decreases with *d*_G_, it suggests juxtacrine signalling (Fig 3C, left). On the other hand, in a collection of cell pairs within a given *d*_G_ range, if *r* decreases with *d*_E_, it suggests paracrine signalling (Fig 3C, right). These two types of signalling can co-exist.

Before moving on to the analysis of signalling mechanisms, we provide further evidence that the proposed method of estimating geodesic distances in a GFP data results in realistic values. Fig 4A shows a montage of x800 EM images of anterior pituitary tissue, which is different from those GFP imaged (D1, D2, D3). In this the cellular contacts between lactotrophes are clearly visible. Although there might have been some shrinkage of the cells [17, 18], since geodesic distance is a scale-invariant property it is expected that such an image will give a realistic estimate of the distribution of geodesic distances between cell pairs. Each lactotroph is shaded in a different colour to distinguish individual cells. There are 18 cells that are fully contained in this montage, and are labelled by a *. For these cells, geodesic distances are calculated, and their distribution is shown in Fig 4B as a histogram. In comparison, geodesic distances in the GFP dataset are calculated for the same number of 18 cells nearest to the tissue centre (see Fig 1C). Their histogram is shown in Fig 4C. Amongst those 18 cells, one was found to be isolated from the other 17 cells, causing the histogram y-axis limit smaller than that in Fig 4B. Otherwise, however, geodesic-distance distributions in Fig 4B and 4C appear to be similar, endorsing the use of the present method of geodesic distance calculations in the GFP datasets. This figure also confirms the tight packing of the cells.

### Juxtacrine signalling underlies the observed space-time correlation

**Fig 5:**
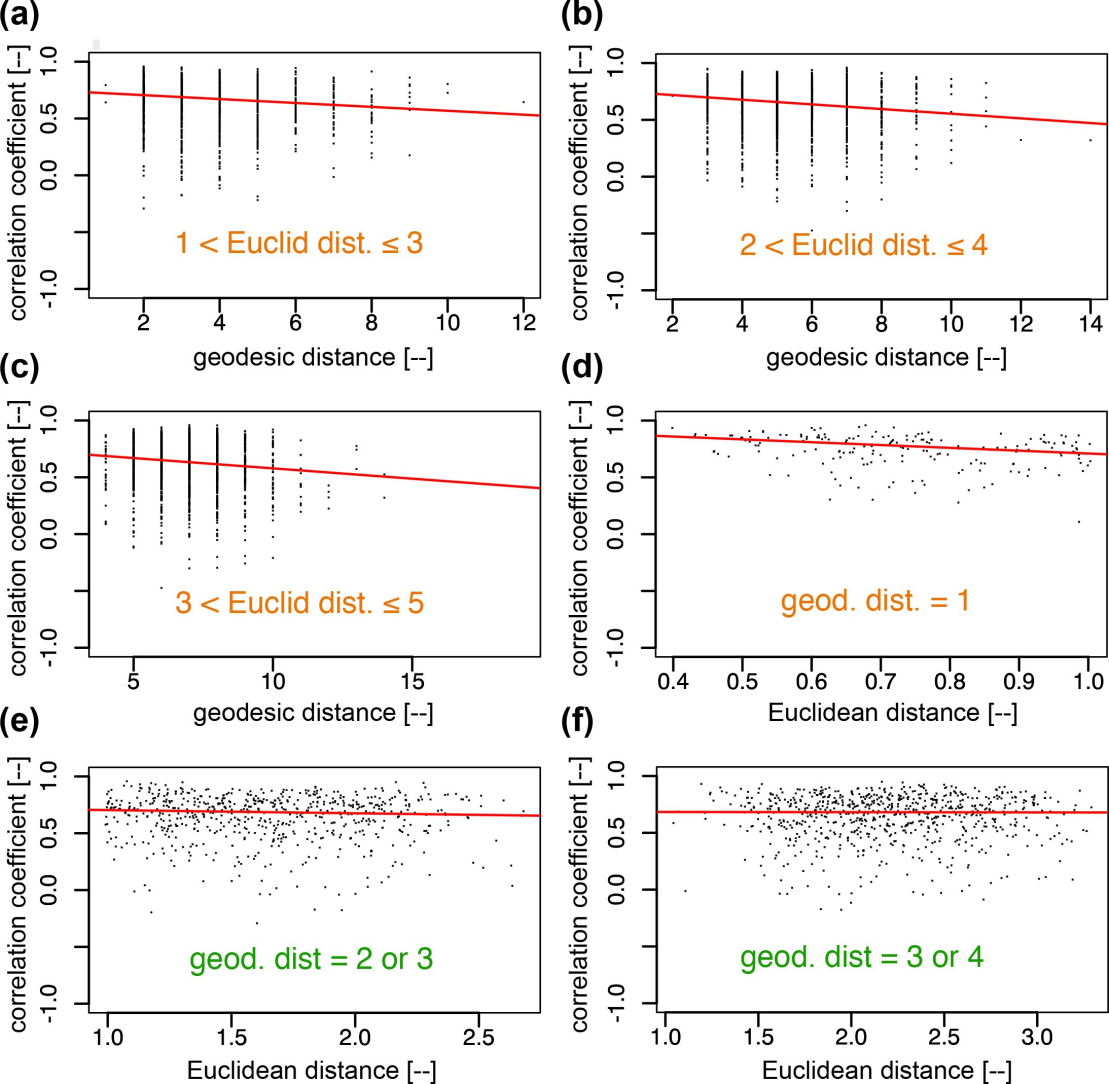
Tests of signalling mechanisms, juxtacrine and paracrine, in the GFP signals. Juxtacrine signalling is tested in **(a)—(c)** with geodesic distance (*d*_G_) in the subset of cell pairs that are within a given Euclidean distances (*d*_E_) range, while paracrine signalling is tested in **(e)—(f)** with Euclidean distances in the subset of cell pairs that have geodesic distances larger than 1. For both signalling modes, a decline to the right in the median regression line suggests the presence of each mode. From the *p*-values (S1 Table) we conclude the dominance of juxtacrine signalling. The cell pairs characterised by *d*_G_ = 1 in **(d)** are pairs of cells in direct contact. The clear decline in their trend line suggests that the juxtacrine signalling is stronger when there is a larger contact area assuming that, for these cell pairs, greater contact area is correlated with smaller Euclidean distance (i.e. if two cells, each assumed to have a ball shape of a similar diameter, are in contact, the shorter the inter-centroid distance, the larger the contact area).

The presence of juxtacrine and paracrine signalling is investigated in the cell-pairs within a limited range of Euclidean (*d*_E_) and geodesic (*d*_G_) distances, respectively, where *d*_E_ and *d*_G_ are set small. This is because small *d*_E_ (*d*_G_) is generally associated with large δ*d*_G_/*d*_G_ (*resp*. δ*d*_E_/*d*_E_, where δ*d*_G_ denotes variation in δ*d*_G_ while δ*d*_E_ variation in *d*_E_), allowing better identification of juxtacrine (*resp*. paracrine) signalling. Fig 5 shows the results of this analysis in dataset D1 using quantile regressions, for juxtacrine (a–c) and paracrine (e, f) signalling mechanisms. Statistical test results are summarised in S1 Table for all three datasets. The trend lines strongly suggest the presence of juxtacrine signalling, with no strong evidence of paracrine signalling (notice that the trend lines in (e) and (f) are much flatter than that in (d), with slopes of (d) -0.25, (e) -0.028, and (f) -0.0018). However, the latter cannot be entirely ruled out as one may conclude the presence of some, albeit weak, paracrine signalling mechanisms if the cell-pair samples in each distance-limited test are pooled across the three datasets (see S1 Table).

Note that in panel (d), unlike (e) and (f), the cell pairs of geodesic distance 1 imply that cells are physically contacting. Hence, the clear trend of decreasing correlation coefficient with Euclidean distance suggests that the more tightly packed the cells the stronger the correlation. This could, for example, be facilitated by gap junctions on the cell surface.

Our conclusions stated above remain consistent when we repeat our analysis based on the cell size distribution identified by confocal microscopy instead (S3 Table).

### Signalling mechanisms appear consistent at the transcriptional level

Having identified juxtacrine signalling in a population of cells in a tissue at the level of expression of GFP reporter signal, we now attempt to approach its origin more directly in the expression of the *transcription rate* of the reporter gene, as a more direct measure of the promoter activity of the prolactin gene regulatory elements contained in the prolactin-d2EGFP transgene. If successful, this analysis will show that the spatial correlation in transcription is mostly due to juxtacrine signalling and therefore provides a more fundamentally mechanistic insight. This approach removes the influence of post-transcriptional activities in GFP expression (Fig 1A).

**Fig 6:**
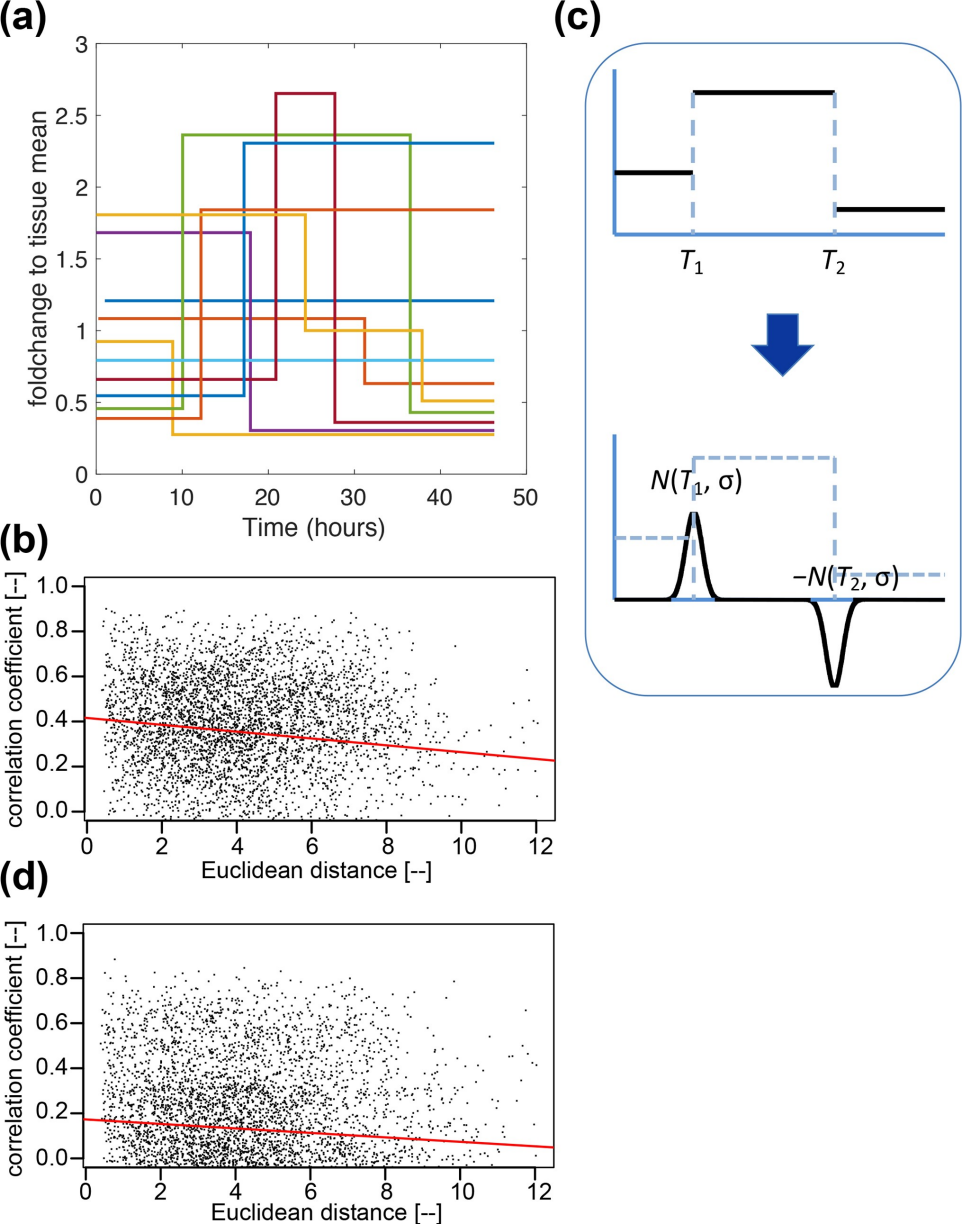
**Analysis of transcriptional profiles inferred by SSM ([7], see also Fig 1(A)). (a)** Transcription profiles of ten chosen cells as in Fig 2A. In the SSM their dynamic behaviour is approximated by step functions that have discrete switch points at times around which the transcription rate is estimated to have changed its value given the reporter data. *A-priori* these rates can take an arbitrary value and are estimated from the reporter data as part of the Bayesian model fitting algorithm described in [7]. **(b)** The correlations defined using the first score function (Sc1) defined in the text decreases as Euclidean distance increases. Results of statistical tests are in S2 Table, which also summarises the results for juxtacrine and paracrine signalling tested in the transcriptional profiles, showing the dominant presence of juxtacrine signalling as seen for the GFP signals (see Fig 5 and S1 Table). **(c)** A different representation of transcriptional activity using the second score function (Sc2) defined in the text which puts more emphasis on the timing of the switches than the level of transcription. Each switch is represented by a signed normal distribution with the same amplitude, centred at the switch time, with the same standard deviation being set to 3 hours in reference to ([8], Fig 2Aii). The drop in transcription rate at time *T*_2_ is reflected by a negative sign. **(d)** Space-time coordination is assessed using median regression of the correlations implied by Sc2 applied to the transformed transcription profiles shown in (c). Scores in these two representations are found to show qualitatively the same behaviour in various signalling tests, as summarised in S2 Table.

As explained in [8] the Stochastic Switch Model (SSM, [7]) enables the inference of different levels of transcription rate as well as the timing of switches between different levels of activity. The model uses a reversible jump Markov chain Monte Carlo algorithm [19] to produce a posterior probability distribution over all possible transcriptional profiles for each cell. Post-processing of this distribution enables the extraction of a number of accepted candidate transcriptional profiles, each with an associated probability of occurrence (See figure 2A in [8])). Consequently, the transcriptional analysis for each cell provides a weighted analysis of all possible transcriptional profiles taking into account the probability of occurrence of each profile (see Materials and Methods for more details). Fig 6A shows the estimated transcription profiles for the ten example cells considered in Fig 2A, which are showing characteristic temporal changes in transcription at a few distinct switch time points [1]. We then perform a space-time analysis, that is analogous to the one applied to the GFP signals (Fig 5), to the distribution of reconstructed transcriptional profiles.

To measure and quantify the correlation between such discrete profiles as in Fig 6A, there are, in theory, a range of possible scoring functions. Here we define two score functions, *Sc1* and *Sc2*, by using Eq 1 in Materials and Methods. To compute Sc1, we take the transcription profiles for each pair of cells weighted by their probabilities and compute the Pearson correlation coefficient. Thus, this score function considers the correlation in the transcription levels over time. To compute the second score, Sc2, we focus on the times of the transitions. In this we replace the transcription profiles by the sum of normal distributions placed at the transition points as illustrated in Fig 6C and measure the Pearson correlation between these. The standard deviation of these is set to 3-hours in accordance with the typical spacing found in the profiles (see [8], Fig 2Aii).

Analysis using both types of score function shows significant space-time correlation at the transcriptional level (Fig 6B and 6D). The analytical approach using Euclidean and geodesic distances is analogous to the approach we developed for the GFP signal above and is summarised in S2 Table. Although it is less clear in individual datasets, when cell-pair samples are pooled across all three datasets, juxtacrine signalling appears to be the dominant mechanism of cellular interaction, with only a weak indication of paracrine signalling. These results are consistent with those found for the GFP signals (S1 Table). The results in S2 Table are for score function Sc1 but use of Sc2 yields qualitatively the same results for the pooled data as shown in S2 Table.

### A stochastic model for juxtacrine coupling

**Fig 7:**
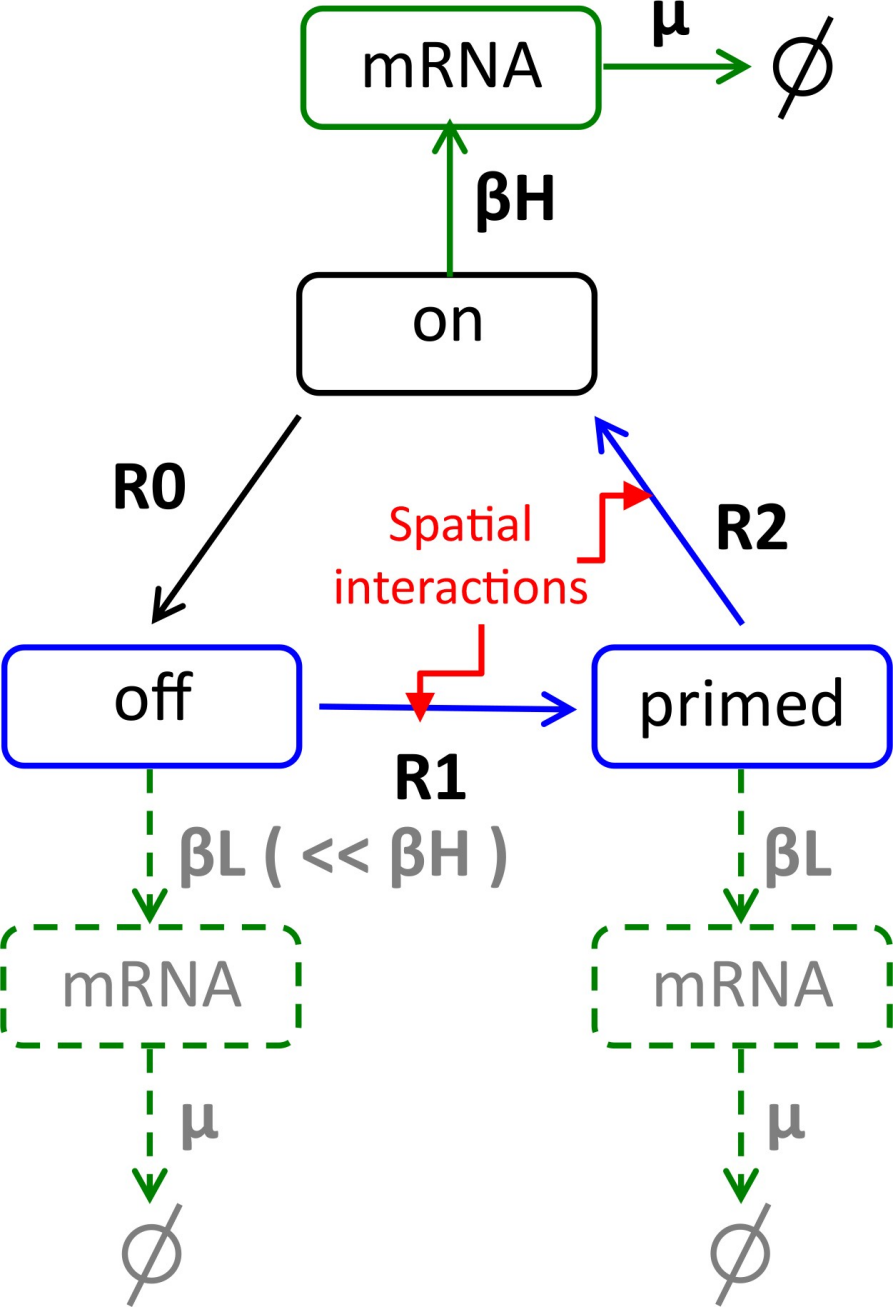
A schematic diagram of the model for the transcriptional dynamics in each cell. The elementary pathway between *on*, *off*, and *primed* is described by a Markov process with an exponential holding time with parameters R_i_ = 1/t_i_. The core of this model is the three cyclic gene states, *on*, *off* and *primed*. The pathways accompanied by *R*_0_, *R*_1_ and *R*_2_ represent the transitions from *on* to *off*, from *off* to *primed*, and from *primed* to *on*, respectively. Conditional on the state we assume that gene expression follows a Markov jump process with birth rates, *β*_H_ for *on* and *β*_L_ for *off* and *primed* states, and degradation rate *μ* for mRNA molecules (same for all three states). The values of the rate parameters are found in Materials and Methods. Spatial coupling is implemented by assuming that the rates *R*_1_ and *R*_2_ are a function of the number of *on* genes in the connected cells (see Eqs (4 and 5) in Materials and Methods).

More details about the model are given in **S1 Text**. We proceed by proposing a stochastic model for prolactin gene expression that incorporates juxtacrine signalling (Fig 7). We address the question of whether such a model with parameters estimated from experimental data can reproduce the observed space-time structure. We assume a three-state model for the prolactin gene as suggested by the analysis in [1], which assumes that each allele can be in one of three states: *on*, *off* or *primed* (Fig 7). When a gene is *off* or *primed* none or very little mRNA is transcribed, while when it is *on*, mRNA is produced at a faster rate. Moreover, the gene transitions from the *off*-state to the *on*-state only occur via the *primed* state and the times in each of these states are exponentially distributed with half-lives *t*_off_, *t*_primed_ and *t*_on_, respectively. The rates and the transcription rates corresponding to the three states are estimated from the data (see Materials and Methods for more details). To model coupling between the cells we assume that a cell senses the states of its neighbours and changes its gene cycle dynamics accordingly. More specifically, here we assume that for a cell the rates of transition between the *off* and *primed* states and between the *primed* and *on*-state are influenced by the number of *on* genes in the connected cells as described in the Materials and Methods.

**Fig 8:**
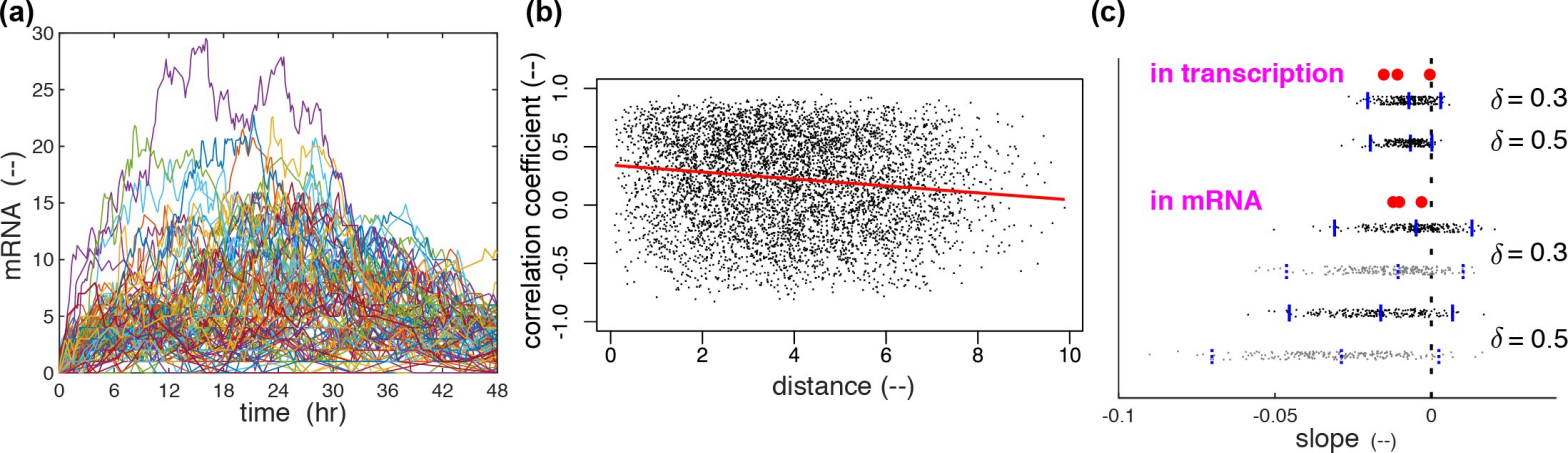
Simulation results and their correlation analysis. **(a)** An example of the mRNA profiles simulated by the model developed above (Fig 7) with parameter values found in Materials and Methods. This set of 108 profiles collectively display a shape that first increases then decreases in time, as in the GFP profiles in experiments (S1 Fig.). **(b)** Relation between pairwise correlation coefficients and Euclidean distance in the simulation shown in (a) at the mRNA level. **(c)** Distributions of the estimated slope coefficients (black dots) between the simulated profiles (transcription-rates and mRNA) and Euclidean distance, over 200 simulations. They are accompanied by the blue bars to represent the respective median and the 95% central interval. The distribution at the transcription-rate level is compared to the slopes obtained with the SSM-inferred transcription-rate profiles from the experimental data (three red blobs), while the distribution at the mRNA level is accompanied by the slopes at the GFP level in the same experiments. The grey dots are of the simulations in which the spatial interactions are in action also in the transcription dynamics as defined in Eq (6). This is one possible mechanism that makes the slope of the correlation coefficient in mRNA number steeper. Other potential mechanisms are discussed in the text.

An example of mRNA profiles of all the cells in one simulation is shown in Fig 8A, where a characteristic mean convex shape is reproduced as observed in the real experiments (S1 Fig). The corresponding correlation analysis is shown in Fig 8B, where, for all cell pairs, correlation coefficients at the mRNA level are plotted against Euclidean distances, with a trend line resulting from the median regression. The negative slope of this trend line suggests space-time correlation as seen above in the analysis of experimental data. The correlation analysis can also be done by using the transcription-rate profiles (*β*(*t*), defined as Eq 10 in Materials and Methods, see S2 Fig for the transcription-rate counterparts of Fig 8A and 8B). The results are comparable to the results in the experimental data at the level of the SSM-inferred transcription-rate profiles (see Fig 6B for dataset D1), while the results in mRNA profiles may indirectly be compared to those of the experimental data at the GFP level. When such a set of simulation and analysis are repeated 200 times, we see that negative values dominate, both at the transcription-rate and mRNA levels, as is shown in the distributions in Fig 8C (signalling and its associated dynamics are highly stochastic, and therefore the slopes could take positive or negative values. The role of signalling is to shift the distribution in the negative direction. S4 Fig shows the slope distribution when cells are not communicating. We also see that the distribution of the slope of the trend line at the transcription-rate level matches nicely the slopes determined in the three experimental datasets. Gradients are greater when we restrict to the short range (<24 μm, or equivalently twice the cell diameter) than in the longer range. This is also true in the experimental data. However, as already implicated in the analysis of the experimental data, the spatial coupling is more likely to be in action also in the processes downstream to the *on*-*off*-*primed* gene cycle up to the GFP profiles. If we assume, for example, an additional mechanism in the transcription dynamics defined by Eq (6), the slopes, on average, become larger as shown by the distribution of the grey dots in Fig 8C. This is one possible mechanism leading to high space-time correlations at the GFP level, while others will be discussed in the Discussion section.

## Discussion

### Technical issues

Space-time coordination in the expression of the prolactin gene was reported for the first time by [8] and the importance of gap junctions for this coordination was discussed. The present study strongly endorses such a picture and uncovers the role of juxtacrine signalling in a robust fashion. In addition, the present study shows a weak contribution of paracrine signalling.

While we have seen that GFP-visible cells were densely packed (Figs 3A and 4 and [20]), it is still a possibility that the network structure was not fully revealed. A particular possibility is that cells are connected in 3D-space, resulting in an overestimation of geodesic distance *d*_G_ when *d*_G_ >2. Geodesic distances can indeed only be overestimated when viewed in 2-D. However, this does not challenge our conclusion that juxtacrine signalling is functioning because if geodesic distances are overestimated the trend line in the plot of correlation coefficients *v*. geodesic distance, will show an even steeper decline. Note also that the influence of juxtacrine signalling is also evident for touching cells, characterised by *d*_G_ = 1, which is free from this bias (Fig 5D). On the other hand, in the test of paracrine signalling, any overestimation of geodesic distance (*d*_G_) would influence the membership of a cell-pair subset, in which the correlation coefficient is regressed on Euclidean distance. In the present study, tests were performed in two overlapping *d*_G_ ranges, 2 or 3 and 3 or 4, so that the general lack of a negative trend suggests possible corrections in the estimation of geodesic distances are less likely to change the conclusion (see S1 and S2 Tables, remember *d*_G_ was set small (see Results)). Nonetheless, a precise 3-D estimation of the cellular network might be able to rule out paracrine signalling with more clarity, and in general this remains an important task for future work for prolactin and other systems. Imperfect cellular segmentation might be happening during image analysis. Overlap of the cells due to boundary errors could cause errors in the estimated, but this is only relevant for touching pairs. Therefore, the influence of this on the trend line in the plot of correlation coefficient against distance should be minor.

In the present work, we used the linear correlation coefficient as a measure of temporal coordination in a pair of time series. This uses a zero time lag between the two time series. Using other time lags tends to decrease correlations (S5 Fig). While this is a natural choice, which appears successful in distinguishing between signalling types, this is not the sole candidate. While we tested two score functions with qualitatively similar results, the use of other score functions may capture different aspects of the system, in particular the temporally discrete nature of the back-calculated transcription activities as a step function (see Fig 6A).

### Biological issues

We have so far established that prolactin gene promoter activity shows space-time correlations on the spatial scale of 100 μm and the temporal scale of 48 hours. Coordination of behaviour across a tissue allows for more accurately controllable hormone production by the tissue as a whole, and may be important for coordination of major episodes of hormone production, such as during the physiological demands of lactation.

We have also established that the observed space-time correlations are mediated by juxtacrine signalling occurring at the level of gene transcription. Stronger evidence for the juxtacrine signalling in the GFP signals than in the inferred transcription rates suggests the involvement of in-/post-transcription juxtacrine signalling. The next question is to reveal the molecular mechanisms that underlie juxtacrine signalling, which may be associated with multiple molecules or channels. Regarding this question, our preceding work has shown that a gap-junction blocker, AGA (Alpha-glycyrrhetinic acid), diminishes the space-time correlation [8]. Application of the methods developed in the present paper to AGA-treated tissues may reveal the loss of juxtacrine signalling in those tissues. However, no cell-diameter data is available to us for the AGA-treated tissue. Such an investigation will need careful determination of cell sizes in a pharmacological environment. Moreover, it should be noted that gap-junction blockers, such as AGA, often have non-specific effects and these may disrupt the network structure. There is a need to specifically delete Cx43 (connexin 43) in PRL cells. Post-transcriptional juxtacrine signalling may also be by mRNA trafficking between cells. A future mathematical model that involves prolactin and other proteins is likely to involve such post-transcription mechanism(s).

While juxtacrine signalling was found to be predominant, it may be too early to rule out the presence of paracrine signalling *in vivo*. This is because paracrine signalling may be impaired in tissue slices, where blood supply and other mechanisms that could influence transport factor are not present.

We have shown in [8] that space-time correlation arises in the course of development, and that such a structure is much less visible in embryonic/neonatal rat tissues. The application of the present analysis method will require careful determination of cell sizes at each developmental stage. If the proportion of GFP-visible cells at embryonic stage is much smaller than at adult stage, cellular network estimation may only be possible by the use of a cellular membrane marker, which makes all cells visible. Including also the case of adults, determining the degree of influence of non-GFP-visible cells remains an important question in future work.

As reviewed briefly in the Introduction, the discovery that the gene expression is stochastic and heterogeneous, while showing some degree of space time coordination was made very recently [8]. The mathematical model developed in the present study is the first model that addresses all three properties, stochasticity, heterogeneity and space-time coordination, with a proper consideration of the refractory period in gene transcription. We have parameterised this model by fitting it to experimental data and shown that it can reproduce the observed space-time correlation. In future this model may be used to guide biological experiments, serving as the base model, with modifications such as the inclusion of more juxtacrine and paracrine signalling channels.

It could be objected that our analysis has not ruled out a situation where a very slowly diffusing molecule that on the relevant time-scale only reached cells neighbouring that which produced. However, this would need a molecule that diffuses less than a few hundred μm over a time scale of a few hours which seems unlikely and in any case such signalling would likely function as though it was true juxtacrine signalling.

In [8] we discussed a global picture where transcription is highly stochastic but has some coordination of bursting at distances up to approximately 35 μm in adult pituitary tissue, but not at greater distances. We hypothesised that short-range signalling allowed uncorrelated behaviour of well-separated cells combined with short range cell-to-cell communication that may stabilise longer term changes in the expression level of the tissue, such as those associated with the oestrus cycle or lactation. Dynamic control is necessary (e.g. acute suppression or activation by hypothalamic regulators) and while the heterogeneity of transcription across cells may well guard against overshooting or oscillation, when making rapid changes local coordination may be necessary to overcome the damping effect of this and allow a quicker response.

### Applications and usability

The overall method developed in the present research is generic and can therefore be applied to a wide range of other systems. For example, they may be applicable to other dynamic processes that may be important for prolactin and other endocrine cell function (e.g. Ca2+, voltage etc.). The good agreement between the results obtained in the GFP signal (S1 Table) and the results obtained at the transcription level (S2 Table) suggests that juxtacrine signalling is predominant.

## Materials and methods

### Ethics statement

Animal studies were performed under UK Home Office licence (PPL: 40/3296 and PPL:70/8082) following local ethical review by the University of Manchester's Animal Welfare and Ethical Review Body.

### Experimental

#### Animal studies

Animals used have been described previously [21]. Animals were sacrificed by a schedule 1 method (exposure to a rising concentration of CO2 followed by cervical dislocation) followed by resection of pituitary glands.

#### Preparation and imaging of pituitary tissue

Preparation, culture and imaging of pituitary tissue were performed as described in [8]. Briefly, pituitaries were resected, washed in HBSS and sliced into 300μm coronal sections using a vibrating microtome (Campden Instruments, UK).

#### Confocal microscopy

Pituitary slices were cultured on 0.4μm millicell filter stages (Merck Millipore, UK) in 35mm glass coverslip based dishes (Greiner Bio-One, UK) with access to medium (DMEM + 4.5 g/l glucose, 10% FBS, 1mM sodium pyruvate, 100 U/ml penicillin, 100 μg/ml streptomycin and 2mM ultraglutamine) and air. Pituitaries were imaged in basal medium for 48h, following which pituitaries were activated with forskolin (5μM) to show that the tissue remained viable in long-term culture. Confocal images were captured using Carl Zeiss laser scanning confocal microscopes (LSM): Pascal, 710 and 780 with a Fluar 10 X magnification 0.5 NA objective and with pituitary tissues cultured at 37C in PeCon XL incubators (PeCon, Germany) with a humidified atmosphere of 95% air and 5% CO2. Excitation of d2EGFP was performed using an argon-ion laser at 488nm with emitted light captured by two detectors simultaneously with different levels of sensitivity through appropriate filters (492-544nm) or a selected portion of the spectrum (492-544nm and 546-611nm), which we have termed high dynamic range (HDR) imaging. All imaging was acquired as z-stacks with fluorescence from tissues analysed from maximum intensity projections using ZEN 2010b (Zeiss, UK) or CellTracker software ([22], (http://www2.warwick.ac.uk/fac/sci/systemsbiology/staff/bretschneider/celltracker/).

#### Lightsheet microscopy

Pituitary slices were placed in 1% low melting temperature agarose dissolved in media (DMEM + 4.5 g/l glucose, 10% FBS, 1mM sodium pyruvate, 100 U/ml penicillin, 100 μg/ml streptomycin and 2mM glutamine), then transferred into a 1mm diameter glass capillary and left to set on ice for 15 minutes. The capillary was then placed into the Lightsheet Z.1 (Zeiss, UK) chamber with the agarose embedded pituitary immersed in media, cultured at 37C with 5% CO2.

Z stack Images were acquired with dual-sided illumination and pivot scanning using a 20x1.0 NA W Plan-Apochromat objective (Zeiss, UK) at Niquest rate or beyond. Excitation of d2EGFP was performed using a 488nm diode laser with a 505-545nm emission filter. Imaging was acquired as z-stacks with fluorescence from tissues analysed from maximum intensity projections using ZEN 2010b (Zeiss, UK).

#### Estimation of cell diameters

Cell diameters were estimated from confocal microscopy imaging by measuring the distance over which the fluorescence signal across a cell was greater than the background signal. Cells were measured in x and y planes and across three different time-points (n = 21 cells). Cell diameters estimated by lightsheet microscopy were also calculated by measuring the distance over which the fluorescence signal across a cell was greater than the background signal (n = 22 cells). While exact estimation of cell diameters is very difficult we only use the the mean and variance in our model and these can be estimated with less error.

### Computational

#### Pearson’s correlation coefficients (r)

$$r=\frac{\sum_{i=1}^{n}(x_{i}-\bar{x})(y_{i}-\bar{y})}{\sqrt{\sum_{i=1}^{n}{\left(x_{i}-\bar{x}\right)}^{2}}\sqrt{\sum_{i=1}^{n}{\left(y_{i}-\bar{y}\right)}^{2}}}$$ (1)

where {*x*_i_} and {*y*_i_} represent the observed activity at time point *i*, *i* = 1,…,*n*, for two cells *x* and *y*, while $\bar{x}$ and $\bar{y}$ denote respective their means.

#### Cell size estimations from a spatial cell distribution

Suppose there are *N* cells. Determine their convex hull, which is the minimum convex polygon that encloses all the *N* cells, of which *N*_ch_ cells play the vertices of this convex hull. Then calculate the convex hull area (*A*_ch_). Since the lower and upper bounds of the average cell area are *A*_ch_/*N* and *A*_ch_/(*N*–*N*_ch_), respectively, the corresponding cell diameters are estimated to be √(*A*_ch_/*N*) and *√*(*A*_ch_/(*N*–*N*_ch_)), respectively, assuming the circle that inscribes the square of the average area models the average cell. The assumption of inscribing circles implies that cells account for 79% of the total area.

#### GFP signal reconstruction (Fig 2B)

Suppose Ch. 1 is more sensitive than Ch. 2, and therefore shows signal saturation to high light intensity. Denote the readings of Ch. 1 and Ch. 2 by *I*^1^ and *I*^2^, respectively. First, by plotting *I*^2^ against *I*^1^, determine the range (*I*^1^_min_, *I*^2^_max_) in which linearity holds between these two channels as *I*^2^ = α + β *I*^1^. Then define the reconstructed signal (*I*) as:

$$I\;=\;I^{1}\;\text{if}\;I^{1}\leq I^{1}{}_{\min}$$ (2)

$$=\;\left(I^{2}-\alpha \right)/\beta \;\text{if}I^{1}>I^{1}{}_{\min}$$ (3)

#### Spatial properties

During the microscopic measurement cells are not stationary and move slightly in space. For each cell, x- and y- coordinates of their centroids are output by CellTracker and we take for the position of that cell its median values in time. We index the cells by *i* = 1,…,*N* (*N* cells) and denote their centroids by (*x*(*i*),*y*(*i*)).

Each cell pair (*i*, *j*) is spatially characterised by two distances, Euclidian *d*_E_(*i*, *j*) and geodesic *d*_G_(*i*, *j*). Euclidean distance is the every-day distance that satisfies the Pythagoras theorem, but is divided in the present study by the experimentally determined mean cell size for better comparison to the geodesic distance. Since cells were not stationary during a microscopic measurement, it is defined, for each cell pair, to be the median in the time course (the standard deviation over time of pairwise distance is small. See its distribution in S3 Fig.).

Geodesic distance depends upon a network structure. To sample the latter we proceed as follows. We use the positions of the cell centroids (*x*(*i*),*y*(*i*)) as described above and sample the cell sizes from the cell size distribution which is taken to be Gamma(*μ*, *σ*) with *μ* and *σ* defined by the mean and standard deviation of cell size found as described above. The cells are assumed to be circular and we regard cells *i* and *j* to be connected if those cells overlap. Geodesic distances are calculated using the resulting network and its adjacency matrix *M*_adj_. The network is sampled 999 times and in this way for each *i* and *j* we get 999 samples of the geodesic distance between cells *i* and *j*. For the final geodesic distance *d*_G_(*i*,*j*) we take the median of these.

Suppose, cells are all equal in size, two distances are equal only when they are connected by linearly aligned intermediate cells. Geodesic distance is usually larger than Euclidean distance, apart from exceptional cases, such as two larger-than-median touching cells.

#### Implementations

For all calculations except quantile regressions, Matlab was used, especially with its Bioinformatics toolbox for geodesic distance calculations. For quantile regressions, R was used with its Quantreg package (*http://cran.r-project.org/web/packages/quantreg/index.html*). In the calculations of t-statistic, which is used for testing the significance of the slope in the distribution of correlation coefficient to distance, bootstrap was used.

### Stochastic switch model (SSM)

As mentioned in [8], the fit of the SSM model was tested through calculation of recursive residuals as a way of comparing the prediction from the model and the observed data (for more information see ([7], appendix G). These showed no departure from the model assumptions, indicating that the switch model fitted the data well.

### Simulation model (excluding parameter values estimation)

#### Spatial structure

Cells are modelled to be a circular disk with a variable diameter sampled from Gamma(*μ*, *σ*) with *μ* = 12.0 and *σ* = 2.2 defined by the mean and standard deviation of cell size in μm. They are randomly allocated in the 2-D square field of 90 μm x 90 μm, according to a 2-D Poisson process with the intensity of 0.0012 μm^−2^. Consequently, about 100 cells are allocated in each simulation. Further, it is assumed that two cells are connected if their centroids are apart less than or equal to the sum of their radii (Fig 3C) giving rise to a cellular network structure in the field.

#### Temporal dynamics

(Fig 7). Each cell is assumed to have four copies of hPRL-d2EGFP transgene ([8], materials and methods, animals). A cellular model consists of five elementary processes, each being represented as a Markov process characterised by its rate constant. Three of those processes collectively model the cyclic transition between gene states: *on* → *off* (*T*_0_), *off* → *primed* (*T*_1_), *primed* → *on* (*T*_2_), where a symbol in a bracket denotes the mean duration, whose inverse defines the rate constant (*R*_x_ | x = {0, 1, 2}). These three processes are accompanied by gene transcription (*β*_L_ or *β*_H_), and mRNA decay (*μ*), where a symbol in a bracket denotes a rate constant. The low transcription (*β*_L_) rate is assigned to the *off* and *primed* gene states, while the high rate (*β*_H_) is to the *on* gene state.

#### Cellular interactions

A cell is modelled to sense the states of its neighbours, and changes its gene cycle dynamics. More specifically, the rates of transition between *off* and *primed* (*R*_1_), and between *primed* to *on* (*R*_2_) are influenced by the number of *on* genes (*n*) in the connected cells in the following way:

$$R_{1}(t)=R_{10}(1+\delta n(t))\;\text{in}\;off\to primed\;\text{pathway}$$ (4)

$$R_{2}(t)=R_{20}(1+\delta n(t))\;\text{in}\;primed\to on\;\text{pathway}$$ (5)

where *R*_10_ and *R*_20_ denote respectively the *R*_1_ and *R*_2_ of an isolated cell, while *δ* the positive coupling strength. In sets of simulations, represented with grey dots in Fig 8C, an additional spatial coupling mechanism is introduced as:

$$\beta_{H}\text{(}t\text{)}=\beta_{H0}(1+\delta_{b}n(t))\;\text{in}\;transcription$$ (6)

This is one potential mechanism that may be in action leading to high space-time correlation in GFP signals.

#### Parameter values

Based on the experimental data (see **Estimations of parameters** below), the rate constants are set in hr^−1^ as follows.

*R*_0_ = (1 / 11.605) / 4 inverse of on period (1/*T*_0_)
*R*_1_ = (1 / 3.5) / 4 inverse of refractory period (1/*T*_1_)
*R*_2_ = (1 / 15.1) / 4 inverse of off period (1/*T*_2_)
*β*_L_ = 0.275 / 4 low transcription rate/gene
*β*_H_ = 4.25 / 4 high transcription rate/gene
*μ* = 0.14095 mRNA degradation rate

where values are the mean of the two corresponding median values in S4 Table, while the dividing factor of 4 in *R*_0_, *R*_1_, *R*_2_, *β*_L_ and *β*_H_ comes from the assumption that each cell has four copies of PRL gene (see **Temporal dynamics** above). The main coupling strength (*δ*) is set to 0.3 or 0.5, while *δ*_b_ to *δ*/10.

#### Adjustment to experimental conditions

The GFP time series show a bell-shape mean behaviour (S1 Fig), suggesting that the experimental system was not in equilibrium during the measurement. To account for this non-homogeneous behaviour, the model is adjusted by multiplying *β*_L_ and *β*_H_ defined above byαexp(−*t*/12) i.e.

$$\beta_{L}\to \alpha \beta_{L}\text{exp(}-t/12\text{)}$$ (7)

$$\beta_{H}\to \alpha \beta_{H}\text{exp(}-t/12\text{)}$$ (8)

Here α is such a normalizing constant that when the both sides of equation are integrated over 48 hours they have the same value (without this modification, GFP signal would goes up first, then stay at an equilibrium level, with no return to zero, unlike the observed real GFP signals). It is also found that at time zero, a majority of the cells show a nearly zero GFP signal. Accordingly, the initial condition is set in all cells as follows.

#(on)=0,#(off)=4,#(primed)=0,#(mRNA)=0 (9)

where #(…) denotes the number of enclosed elements.

#### Simulations and correlation analysis

Two hundred independent simulations are run modifying the Gillespie algorithm to allow for the fact that the transcription rate is time-dependent because of the adjustment in Eqs (7 and 8). The time τ to the next reaction at time *t* is calculated to satisfy

$$\log U+\int_{t}^{t+\tau }r(s)ds=0$$

where *U* is a random number drawn from the uniform distribution on (0, 1), *r*(*s*) the total propensity function, unlike in the standard Gillespie algorithm where τ is calculated to satisfy logU+τr(t)=0. Note that the first equation is reduced to the second, when *r*(*t*) is homogeneous.

Each simulation time is set to 48 hours as the same length as GFP time series recorded in experiments. After each simulation, linear correlation coefficients are calculated for all cell pairs between mRNA profiles, or between transcription profiles (*β*(t)) defined as

$$\beta (t)=\beta_{H}N(t)+\beta_{L}(F(t)+P(t))$$ (10)

where *β*_H_ and *β*_L_ are respective high and low transcription rates, while *N*(*t*), *F*(*t*), and *P*(*t*) denote respectively the number of *on*, *off*, and *primed* genes. Such correlation coefficients are analysed by median regression to Euclidean distance between a cell pair, yielding a trend line for each simulation. Its negative slope implies the presence of space-time correlation.

### Estimations of parameters in the model

The parameter values were estimated by analysing the transcription profiles statistically inferred by SSM to two GFP-time-course datasets (male rats with no stimulation; maleCont, maleCont16), and shown in S4 Table. The maximum likelihood estimates of refractory and off periods, *T*_1_ and *T*_2_, were obtained by fitting the sum of two exponential distributions to the durations after a down switch with the assumption of *T*_1_ < *T*_2_, while on period, *T*_0_, was estimated by fitting an exponential distribution to the durations after an up switch. Both durations in the SSM results were analysed as right-censored ones. These period estimations are detailed below. Low and high rates, *β*_L_ and *β*_H_, are the respective transcription rates after a down and up switch.

#### *Maximum likelihood estimation of on* (*T*_0_), *refractory* (*T*_1_), *and off* (*T*_2_) *periods*

In the following, capital *T* denotes a period parameter, while small *t* its realisation in the SSM-generated Markov chain, which represents the posterior distribution of the parameters. An inter-switch duration (*t*) is called complete if it is flanked by two switches, but right-censored if it is open-ended.

Preprocessing. In the continuous SSM, it is often the happening that a down or up switch is followed by the same-direction switch. To fit the SSM result into the current discrete on-off-primed model, such consecutive durations after same-direction switches are merged to form a single inter-switch duration.

On period estimation. As a Markov process with an exponential holding time, *on* period *t*_0_ follows an exponential distribution *t*_0_ ~ *Exp*(−λ_0_ *t*), where λ_0_ denotes the inverse of *T*_0_. By denoting its probability density and distribution functions by *f*_u_ and *F*_u_, respectively, the likelihood function for given sets of complete and censored *on* durations {*t*^comp^_*i*_} and {*t*^cens^_j_} is given as follows.

$$l(T_{0})=\Sigma_{i}f_{u}\text{(}t^{comp}{}_{i};T_{0}\text{)}+\Sigma_{i}(1–F_{u}(t^{cens}{}_{i};T_{0}))$$ (11)

Then *T*_0_ is estimated as argmax *l*(*T*_0_).

Refractory and off period lengths estimation. As Markov processes with an exponential holding time, refractory and off periods, *t*_1_ and *t*_2_, follow respective exponential distributions, *t*_1_ ~ *Exp*(−λ_1_ *t*) and *t*_2_ ~ *Exp*(−λ_2_ *t*), where λ_1_ and λ_2_ denote respectively the inverse of *T*_1_ and *T*_2_. If λ_1_ ≠ λ_1_ is assumed, the probability density function (*f*_d_(*t*_d_)) of the sum of those two random variables, *t*_d_ = *t*_1_ + *t*_2_ is given as follows.

$$f_{\text{d}}\text{(}t_{\text{d}}\text{)}=\lambda_{1}\lambda_{2}/{(\lambda }_{2}-\lambda_{1}\text{)}(exp(-\lambda_{1}t)–exp(-\lambda_{2}t))$$ (12)

while its corresponding distribution function is given as follows.

$$F_{\text{d}}\left(t_{\text{d}}\right)=1/{(\lambda }_{2}-\lambda_{1}{)\{\lambda }_{2}(1-\text{exp}(-\lambda_{1}t))–\lambda_{1}\text{(}1-\text{exp}(-\lambda_{2}t))\}$$ (13)

For given sets of complete and censored duration data, {*t*^comp^_*i*_} and {*t*^cens^_j_}, a logarithmic likelihood is given as follows.

$$l(T_{1},T_{2})=\Sigma_{i}f_{d}\text{(}t^{comp}{}_{i};T_{1},T_{2}\text{)}+\Sigma_{i}(1-F_{d}(t^{cens}{}_{i};T_{1},T_{2}))$$ (14)

With the assumption of *T*_1_ < *T*_2_, *T*_1_ and *T*_2_ are simultaneously estimated as argmax *l*(*T*_1_, *T*_2_).

### Experimental data used in parameter estimations (maleCont & maleCont16)

#### Animals

The generation and characterisation of (hPRL-d2EGFP.F344)455 male rats were described in [21] and the maintenance of the rats was described in [8]. Animals were sacrificed on the morning of the experiment by a rising concentration of CO_2_ followed by cervical dislocation. Pituitary glands were removed, weighed and placed directly into medium (DMEM without phenol red).

#### Preparation and culture of pituitary tissue

Pituitaries removed from transgenic rats, were washed in medium (DMEM without phenol red) and sliced to 250μm thickness in the coronal orientation using a vibrating microtome (Campden Instruments, UK). Pituitary slices were placed on top of a Millicell (Merck Millipore, UK) 0.4μm sterilised culture plate insert filter, positioned in a 35mm glass-coverslip-based dish (Greiner Bio-One, UK) containing 1.3 ml primary cell culture media (DMEM supplemented with 4.5 g/l glucose, 10% Dextran-Charcoal treated FBS (Perbio Scientific, UK), 50 μM Sodium Pyruvate, 0.1μM Ultraglutamine and 500U Penicillin/ Streptomycin. Plates were sealed with a Breathe-Easy® air permeable membrane (Sigma-Aldrich, UK).

#### Time-lapse confocal fluorescence microscopy

Tissue slices were imaged using laser scanning microscopes (Carl Zeiss, UK): LSM780, LSM Pascal or LSM Excitor, maintained at 37C, 5% CO_2_ with humidity. Fluorescent images were taken with a Fluar 10X 0.5NA air objective (Carl Zeiss, UK). For time-lapse imaging, Z-stacks were collected every 15 min and were subsequently rendered into a single image maximum intensity projection for analyses. After 48h in basal culture medium, forskolin (5μM) was added to the dish to stimulate prolactin gene expression and confirm that pituitary slices remained viable in culture over extended time periods. Fluorescence intensity measurements from single cells were generated using CellTracker software v.0.1 (see above for details).

## Supporting information

S1 Fig GFP profiles. Profiles in three experimental datasets show characteristic convex mean profiles with nearly zero signal at time zero.
(TIF)

S2 Fig Simulated transcription-rate levels and their correlation analysis. **(a)** An example of transcription rate profiles simulated by the model (Fig 7). **(b)** Relation between pairwise correlation coefficients and Euclidean distance in the simulation shown in (a) at the transcription-rate level.
(TIF)

S3 Fig Histogram of the standard deviation over time of pairwise distance in dataset D1. (TIF)

S4 Fig Distribution of the slope in the plot of correlation coefficient to Euclidean distance. Correlation coefficients are calculated between mRNA time series simulated by the stochastic model with varying δ, while δb is held fixed at 0. The probability of getting a slope less than zero is marked. The KS p-values for differentiating these distributions are p = 6.3392e-07 (δ = 0.3 vs δ = 0) and p = 2.6551e-12 (δ = 0.5 vs δ = 0.3).
(TIF)

S5 Fig Distribution of the optimal lag. The question arose as to whether one should calculate the correlation for a pair of cells allowing for a time lag. To test whether this was appropriate, we took all cell pairs whose distance is less than or equal to the mean cell diameter and calculated the lag that optimised the correlation. We found that zero lag strongly dominated (148 cells out of 213 had zero lag and 188 of these cells had an absolute lag less than 3h). Dataset D1 is examined.
(TIF)

S1 Table Summary of the signalling type analysis in GFP signals. In 3 replicated datasets, both individual (D1, D2, D3) and combined (DA).
(TIFF)

S2 Table Summary of the signalling type analysis in transcription profiles. In 3 replicated datasets, both individual (D1, D2, D3) and combined (DA). In the second row of DA, scores are calculated in the switch-train representation defined in Fig 6C. Figures are otherwise calculated between the transcription profiles illustrated in Fig 6A.
(TIFF)

S3 Table Summary of cell-size sensitivity in signalling type analysis in GFP signals. In 3 replicated datasets, both individual (D1, D2, D3) and combined (DA).
(TIFF)

S4 Table Parameter values estimated in the SSM results of the two datasets. Except the coupling strength (δ) and the number of gene copies, which are in the main text.
(TIF)

S1 Text A concise and full description of the temporal dynamics of the model. (PDF)
