# Supplementary figures and images for: Disentangling juxtacrine from paracrine signalling in dynamic tissue
Source: PLoS Comput Biol. 2019 Jun 13;15(6):e1007030. doi: 10.1371/journal.pcbi.1007030 (PMC6592563; doi:10.1371/journal.pcbi.1007030)

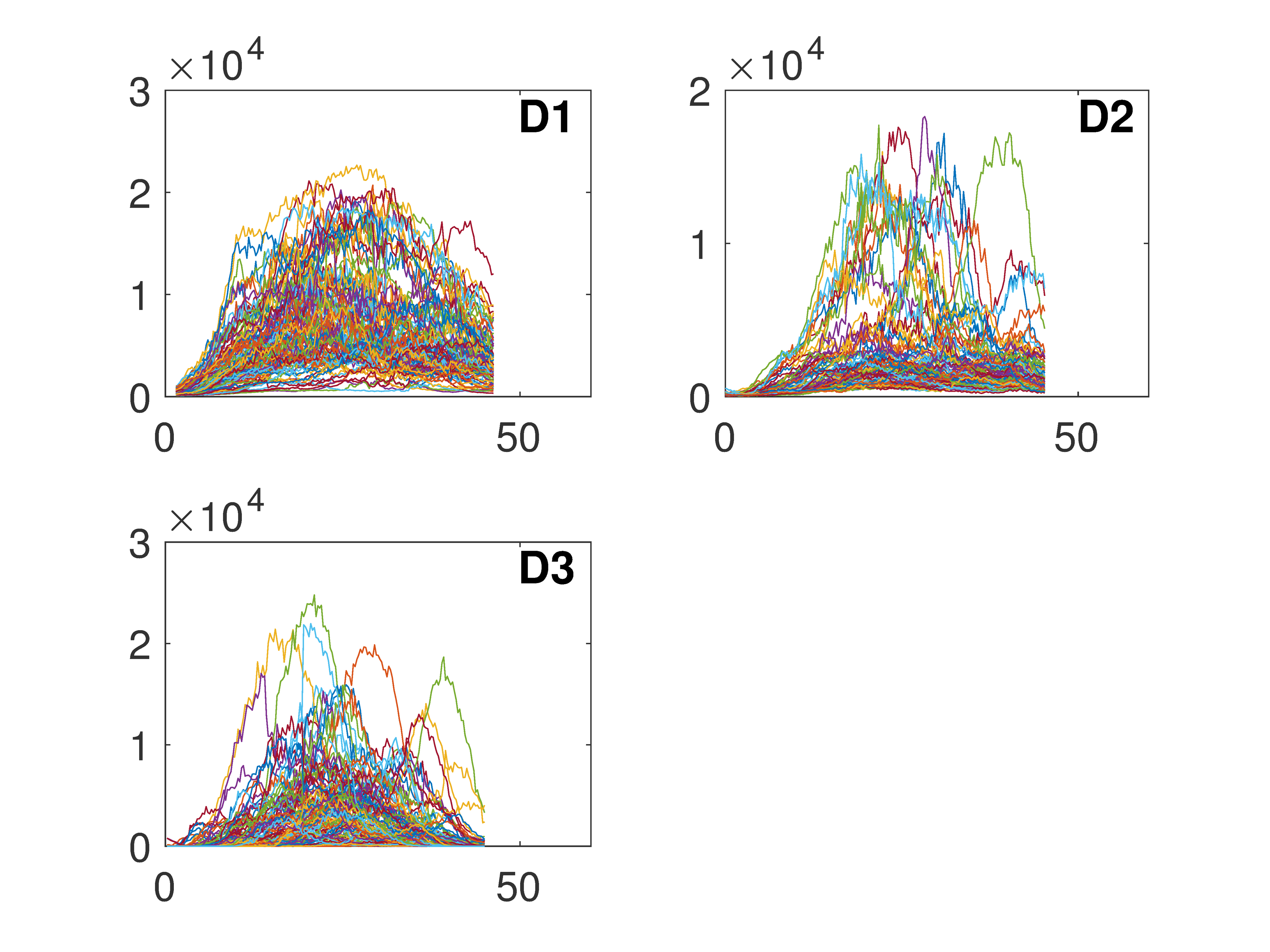

Supplement: S1 Fig — Profiles in three experimental datasets show characteristic convex mean profiles with nearly zero signal at time zero. (TIF) [file pcbi.1007030.s001.tif]

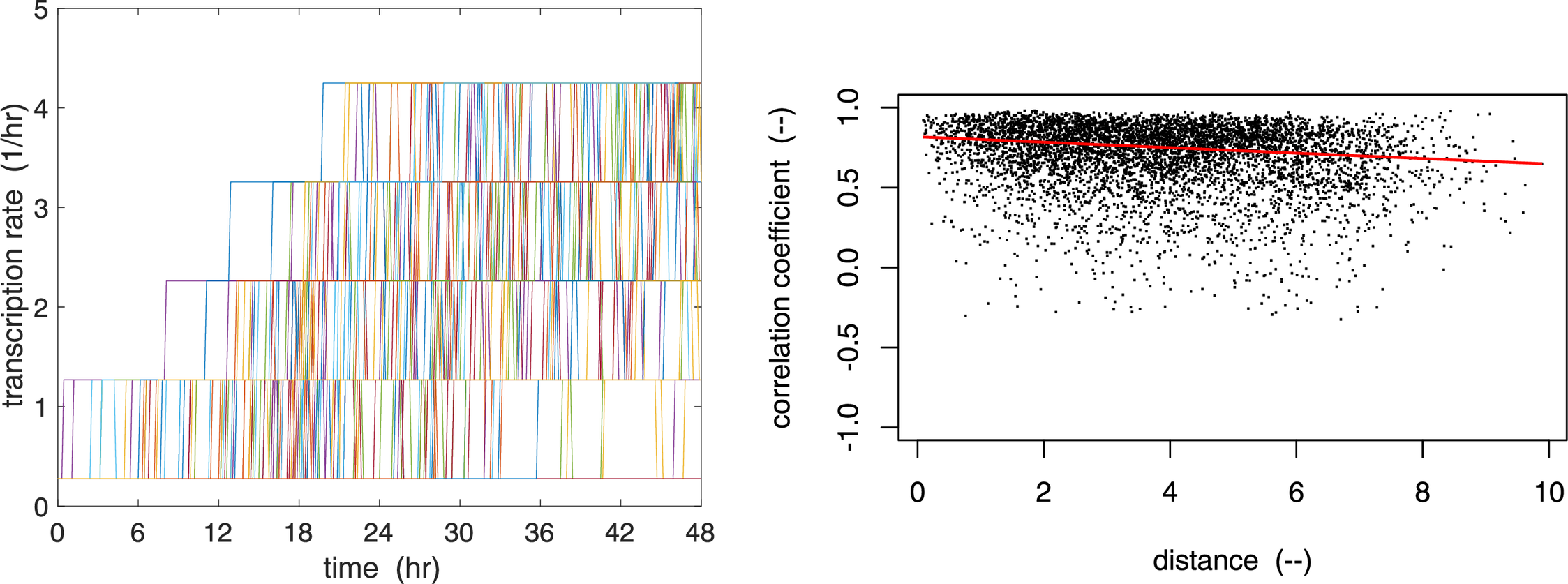

Supplement: S2 Fig — (a) An example of transcription rate profiles simulated by the model (Fig 7). (b) Relation between pairwise correlation coefficients and Euclidean distance in the simulation shown in (a) at the transcription-rate level. (TIF) [file pcbi.1007030.s002.tif]

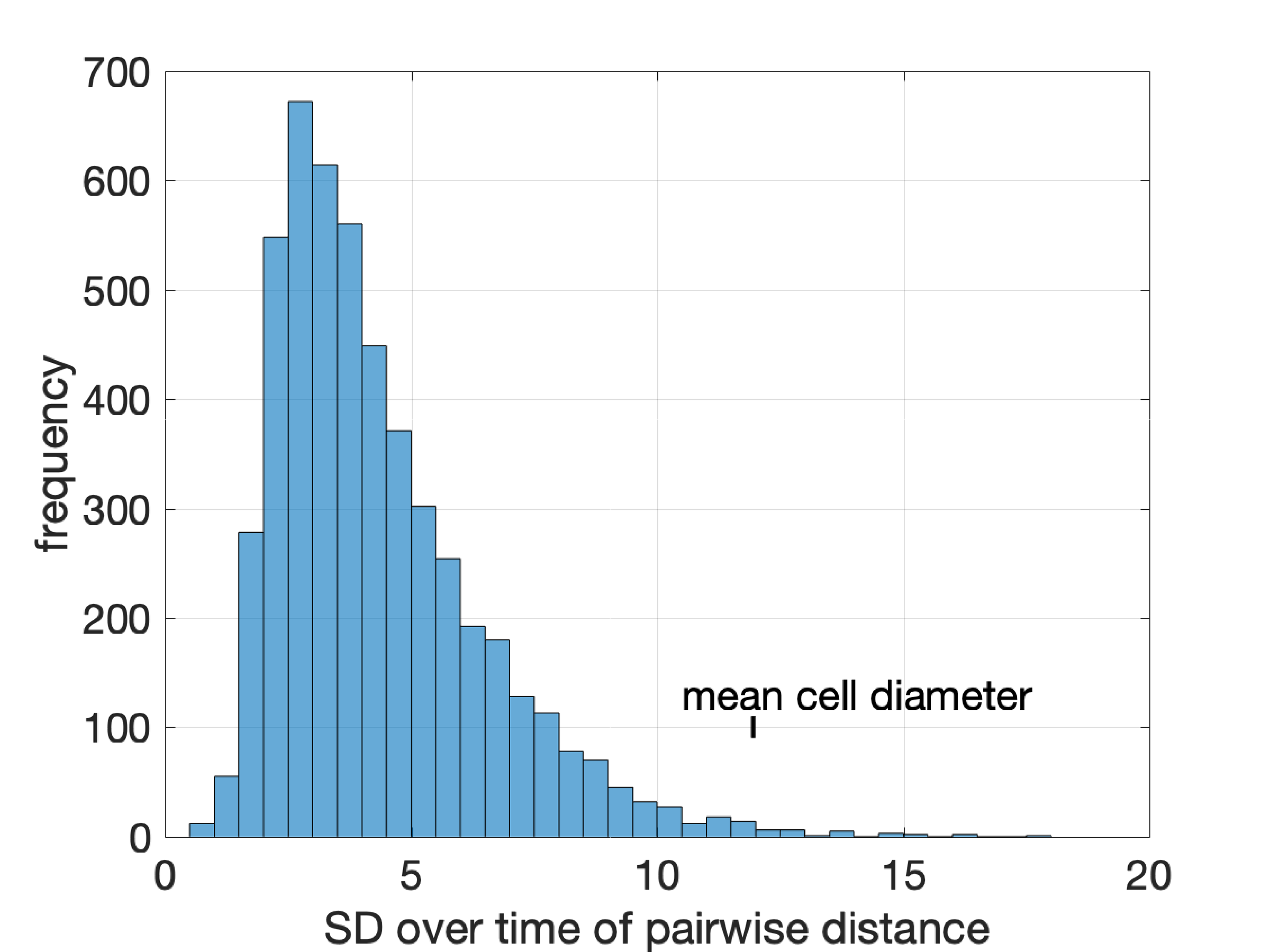

Supplement: S3 Fig — (TIF) [file pcbi.1007030.s003.tif]

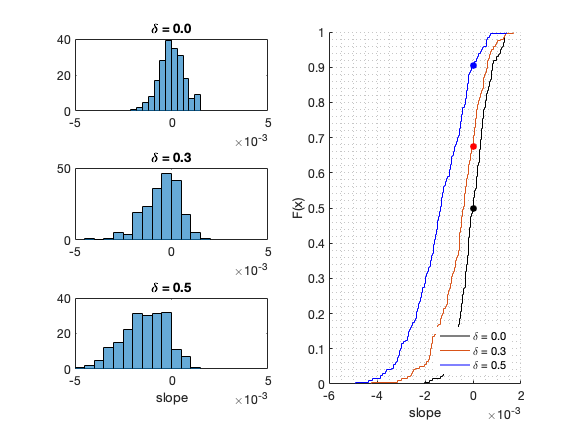

Supplement: S4 Fig — Correlation coefficients are calculated between mRNA time series simulated by the stochastic model with varying δ, while δb is held fixed at 0. The probability of getting a slope less than zero is marked. The KS p-values for differentiating these distributions are p = 6.3392e-07 (δ = 0.3 vs δ = 0) and p = 2.6551e-12 (δ = 0.5 vs δ = 0.3). (TIF) [file pcbi.1007030.s004.tif]

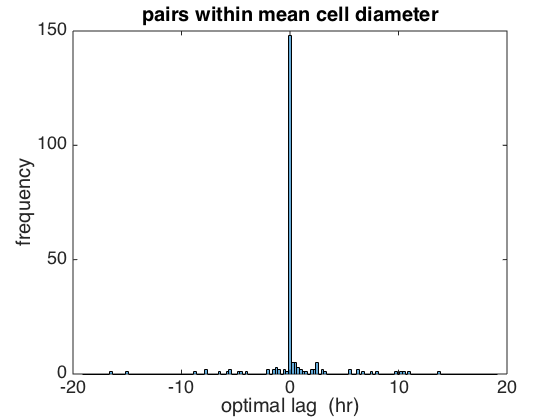

Supplement: S5 Fig — The question arose as to whether one should calculate the correlation for a pair of cells allowing for a time lag. To test whether this was appropriate, we took all cell pairs whose distance is less than or equal to the mean cell diameter and calculated the lag that optimised the correlation. We found that zero lag strongly dominated (148 cells out of 213 had zero lag and 188 of these cells had an absolute lag less than 3h). Dataset D1 is examined. (TIF) [file pcbi.1007030.s005.tif]

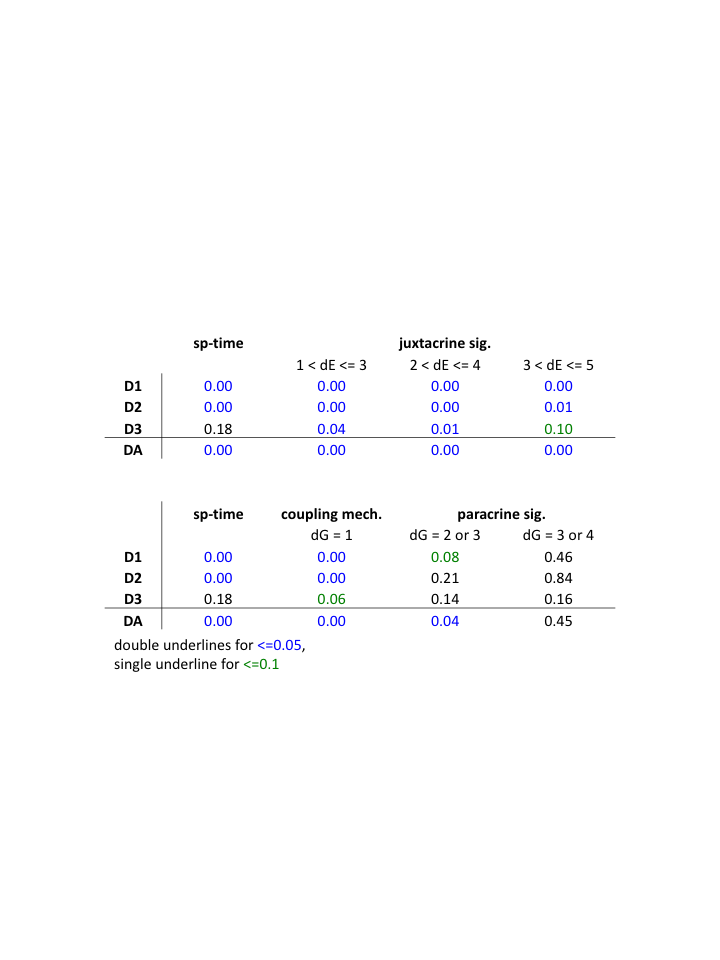

Supplement: S1 Table — In 3 replicated datasets, both individual (D1, D2, D3) and combined (DA). (TIFF) [file pcbi.1007030.s006.tiff]

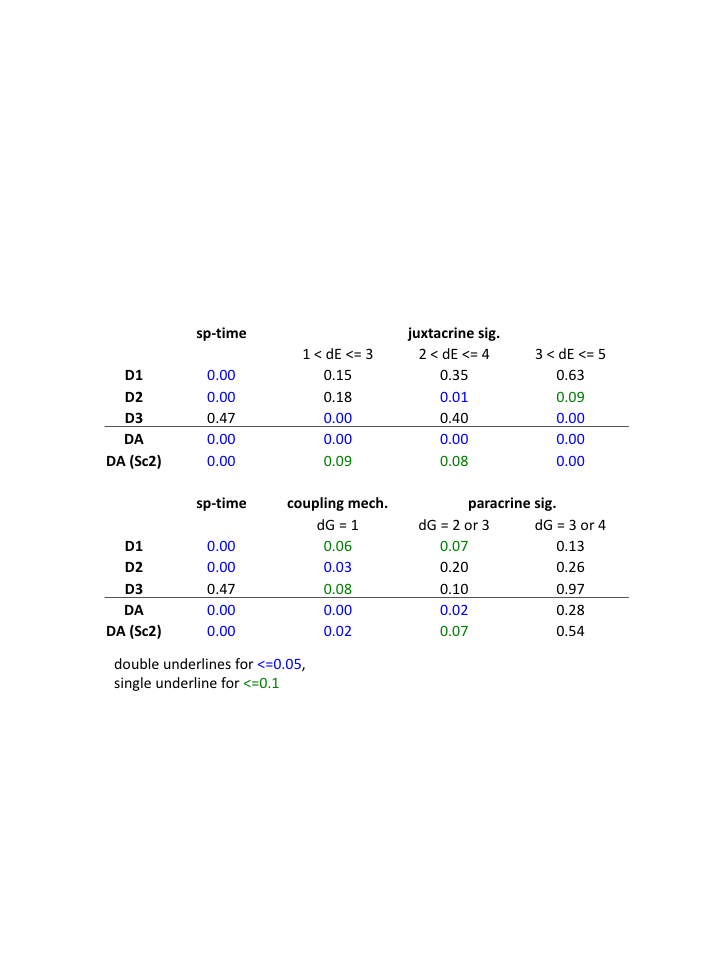

Supplement: S2 Table — In 3 replicated datasets, both individual (D1, D2, D3) and combined (DA). In the second row of DA, scores are calculated in the switch-train representation defined in Fig 6C. Figures are otherwise calculated between the transcription profiles illustrated in Fig 6A. (TIFF) [file pcbi.1007030.s007.tiff]

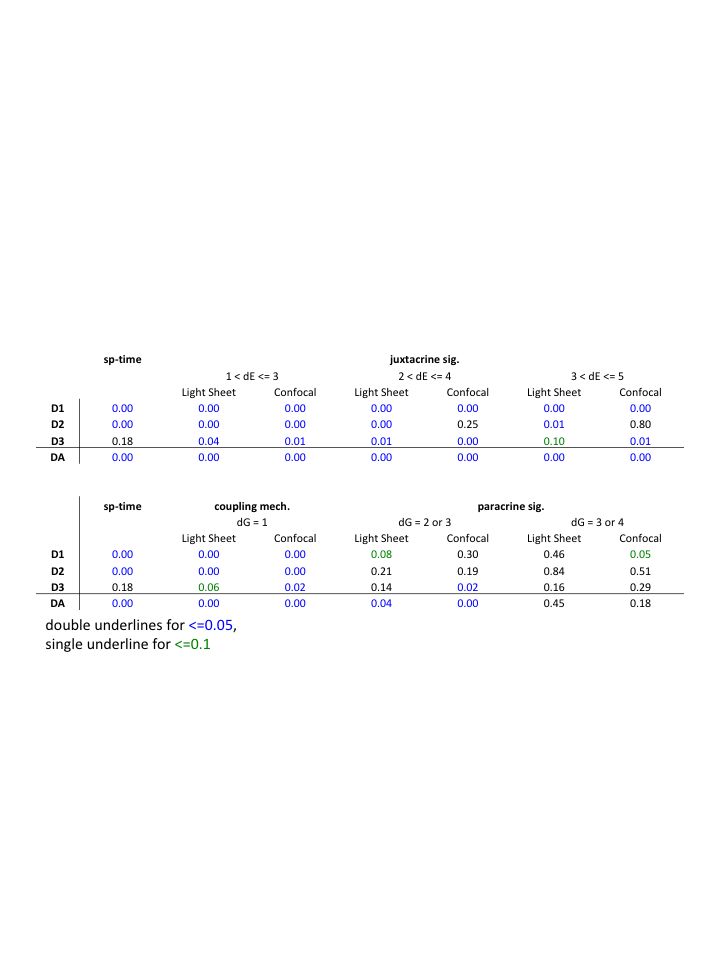

Supplement: S3 Table — In 3 replicated datasets, both individual (D1, D2, D3) and combined (DA). (TIFF) [file pcbi.1007030.s008.tiff]

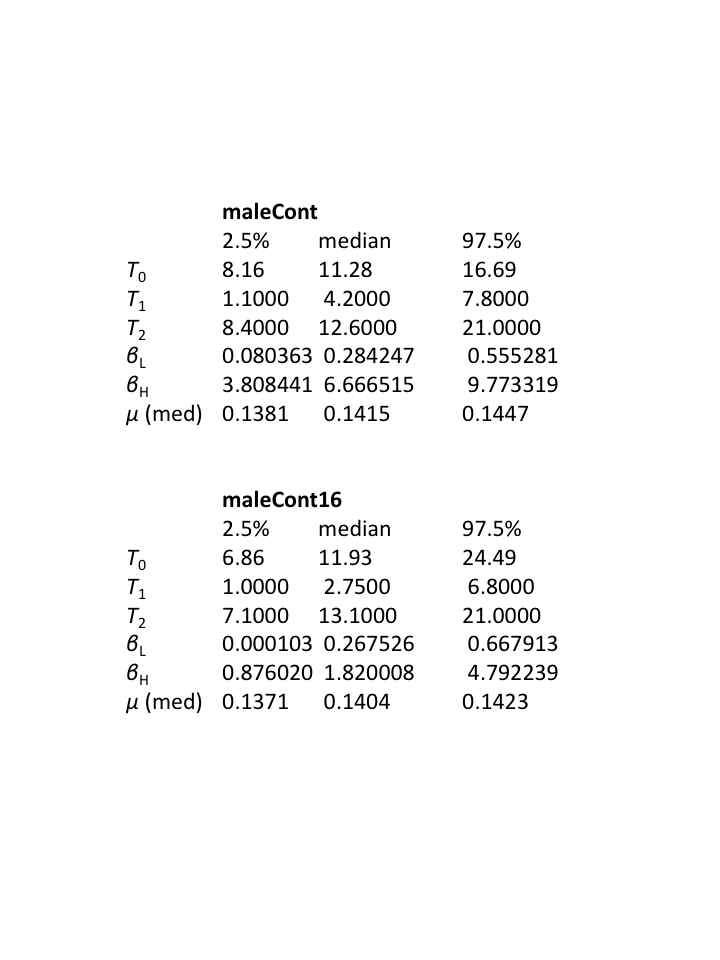

Supplement: S4 Table — Except the coupling strength (δ) and the number of gene copies, which are in the main text. (TIF) [file pcbi.1007030.s009.tif]
