# Supplementary material for: Disentangling juxtacrine from paracrine signalling in dynamic tissue
Source: PLoS Comput Biol. 2019 Jun 13;15(6):e1007030. doi: 10.1371/journal.pcbi.1007030 (PMC6592563; doi:10.1371/journal.pcbi.1007030)
Supplement: S1 Text — (PDF) [file pcbi.1007030.s010.pdf]

Mathematically concise summary of the stochastic model

The information below is sufficient to code the model. Read with the contents in the text.

Spatial structure. Cells are modelled to be a circular disk with a random diameter (D) sampled from a gamma distribution with the parameter values of mean = 11.95 μm and standard deviation = 2.15μm. They are randomly allocated in the 2-D square field of 90 μm x 90 μm, according to a 2-D Poisson process with the intensity of 0.0012 μm^{-2}. Consequently, about 100 cells are allocated in each simulation. Further, it is assumed that two cells i and j are connected if their centroids are apart less than or equal to (Di + Dj) / 2 (Fig 3a), giving rise to a cellular network structure in the field.

Temporal dynamics including cellular coupling (Fig. 6). Each cell has the same temporal-dynamics model that involves 4 molecular species, 5 reactions and 8 parameters. The 4 species are: *on* gene ($n1$), *off* gene ($n2$), *primed* gene ($n3$), and mRNA ($n4$), where $n1$, $n2$, $n3$, $n4$ in brackets denote the respective molecular numbers at time t in hr. At $t = 0$, they are set to: $n1 = 0$, $n2 = 4$, $n3 = 0$, and $n4 = 0$. The 5 reactions are: transition from *on* to *off* ($a1$), transition from *off* to *primed* ($a2$), transition from *primed* to *on* ($a3$), mRNA production ($a4$), and mRNA degradation ($a5$), where $a1$, $a2$, $a3$, $a4$, $a5$ in brackets denote the respective propensities detailed below. The 8 parameters, which are involved in propensity calculations, are: transition rates from *on* to *off* ($k0$), from *off* to *primed* ($k1$), from *primed* to *on* ($k2$); respective low and high transcription rates (bL, bH); mRNA degradation rate (μ); cellular coupling constant (δ); and the normalizing constant associated with bL and bH (Cint). Cells are coupled at the loci of gene-state changes: *off* to *primed* and *primed* to *on*.

The associated stoichiometric matrix (dn) is reads:

	1	2	3	4	5	% react. nb.	
dn = [	−1	0	1	0	0	% on	($n1$)
	1	−1	0	0	0	% off	($n2$)
	0	1	−1	0	0	% primed	($n3$)
	0	0	0	1	−1]	% mRNA	($n4$)

while the propensity in each reaction is given as:

(React. 1) $a1 = k0 * n1$
(React. 2) $a2 = k1 * (1 + \delta * x) * n2$
(React. 3) $a3 = k2 * (1 + \delta * x) * n3$
(React. 4) $a4 = (bL * (n2 + n3) + bH * n1) * (48/Cint) * \exp(-t/12)$
(React. 5) $a5 = \mu * n4$

where x is the number of *on* genes in connected cells, while Cint is the normalizing constant defined by the integral of $\exp(-x/12)$ over $t \in (0, 48)$. Therefore, the transition rates in Reactions 2 and 3 are state-dependent, while transcription rates in Reaction 4 are state-dependent, yet temporally decreasing.

Parameter values, other than Cint mentioned above, are as follows: k0 = 0.0215, k1 = 0.0714, k2 = 0.0195, bL = 0.0688, bH = 1.0625, μ = 0.1409, δ = 0.3.
